# The biophysical properties of TRIC-A and TRIC-B and their interactions with RyR2

**DOI:** 10.1085/jgp.202113070

**Published:** 2023-09-26

**Authors:** Jianshu Hu, Elisa Venturi, Charalampos Sigalas, Takashi Murayama, Miyuki Nishi, Hiroshi Takeshima, Rebecca Sitsapesan

**Affiliations:** 1 Department of Pharmacology, University of Oxford, Oxford, UK; 2 Department of Cellular and Molecular Pharmacology, Graduate School of Medicine, Juntendo University, Tokyo, Japan; 3 Department of Biological Chemistry, Graduate School and Faculty of Pharmaceutical Sciences, Kyoto University, Kyoto, Japan

## Abstract

Trimeric intracellular cation channels (TRIC-A and TRIC-B) are thought to provide counter-ion currents to enable charge equilibration across the sarco/endoplasmic reticulum (SR) and nuclear membranes. However, there is also evidence that TRIC-A may interact directly with ryanodine receptor type 1 (RyR1) and 2 (RyR2) to alter RyR channel gating. It is therefore possible that the reverse is also true, where the presence of RyR channels is necessary for fully functional TRIC channels. We therefore coexpressed mouse TRIC-A or TRIC-B with mouse RyR2 in HEK293 cells to examine if after incorporating membrane vesicles from these cells into bilayers, the presence of TRIC affects RyR2 function, and to characterize the permeability and gating properties of the TRIC channels. Importantly, we used no purification techniques or detergents to minimize damage to TRIC and RyR2 proteins. We found that both TRIC-A and TRIC-B altered the gating behavior of RyR2 and its response to cytosolic Ca^2+^ but that TRIC-A exhibited a greater ability to stimulate the opening of RyR2. Fusing membrane vesicles containing TRIC-A or TRIC-B into bilayers caused the appearance of rapidly gating current fluctuations of multiple amplitudes. The reversal potentials of bilayers fused with high numbers of vesicles containing TRIC-A or TRIC-B revealed both Cl^−^ and K^+^ fluxes, suggesting that TRIC channels are relatively non-selective ion channels. Our results indicate that the physiological roles of TRIC-A and TRIC-B may include direct, complementary regulation of RyR2 gating in addition to the provision of counter-ion currents of both cations and anions.

## Teaser

Hu et al. demonstrate that, independent of other countercurrents, TRIC-A and TRIC-B differentially modulate RyR2 gating. Results also suggest that TRIC-A and TRIC-B are permeable to both K^+^ and Cl^−^, thus enabling them to provide adaptable countercurrents for many cell types.

## Introduction

The trimeric intracellular cation channels (TRIC), of which there are two types, TRIC-A and TRIC-B (Yazawa et al., 2007), are located in sarco/endoplasmic reticulum (SR) and nuclear membranes. Crosslinking studies and electron microscopy indicated that TRICs are a novel type of ion channel that are trimeric in structure (Yazawa et al., 2007). TRIC-B was shown to be expressed at low levels in most cells while TRIC-A was found in high concentrations in cardiac and skeletal muscles and in the brain. This first report of these novel proteins found that the incorporation of purified TRIC-A into artificial membranes led to the appearance of K^+^ and Na^+^ conductances and gave rise to the idea that TRICs were cation channels.

An important role of cardiac and skeletal muscle SR is the storage and release of Ca^2+^ in the process of excitation–contraction (EC) coupling. Ca^2+^ release occurs primarily through RyR1 channels in the skeletal muscle and through RyR2 in the cardiac muscle (Rios and Brum, 1987; Näbauer et al., 1989; Takeshima et al., 1989; Nakai et al., 1990). To maintain a large and rapid release of SR Ca^2+^ during EC coupling, a mechanism is required to prevent accumulation of negative charge within the SR, otherwise the reversal potential (E_rev_) for Ca^2+^ would be rapidly reached, thus disestablishing physiological Ca^2+^ release. Modeling studies indicate that while RyR channels may provide a proportion of their own counter-ion current, other counter-ion pathways are required (Zsolnay et al., 2018). Thus TRICs, as K^+^ channels, would be expected to play a sizeable role.

There is now emerging evidence that TRIC-A, in addition to providing monovalent cation flux during SR Ca^2+^ release, may also interact directly with RyR channels to modulate gating (El-Ajouz et al., 2017; Zhou et al., 2020). El-Ajouz et al. (2017) found that deletion of the *Tric-a* gene in mice led to altered single-channel behavior of RyR1. The open probability (Po) of RyR1 channels derived from the skeletal muscle of *Tric-a* knockout (KO) mice, in the presence of key physiological regulators, was markedly lower than that of RyR1 channels from WT mice suggesting that TRIC-A may be a RyR1 binding protein with a vital role in priming RyR1 for activation. More recently, Zhou et al. (2020), also utilizing *Tric-a* KO mice, suggested that TRIC-A stimulates RyR2 directly. They also found evidence that the flexible carboxyl-terminal tail (CTT) portion of TRIC-A could activate RyR2 whereas the CTT of TRIC-B could not and suggested that TRIC-A binds directly to RyR2 via the CTT domain. In the current study, we therefore sought to further investigate the idea that TRIC-A, but not TRIC-B, directly modulates RyR2 channel function. We expressed RyR2 in HEK293 cells and incorporated mixed membrane vesicles from these cells into planar phospholipid bilayers to monitor the conductance and gating behavior of RyR2 channels that had not been exposed to detergents or the presence of TRIC channels. We then compared the behavior of these RyR2 channels to those that had been coexpressed with either TRIC-A or TRIC-B.

Great progress has been made in determining the structural features of various prokaryotic and eukaryotic TRIC channels (Kasuya et al., 2016; Yang et al., 2016; Ou et al., 2017; Su et al., 2017; Wang et al., 2019); however, the characterization of the functional properties of these unusual ion channels has lagged behind. Purified TRIC proteins have always been reported to be permeable to K^+^ but there has not been a rigorous examination of their permeability or conductance properties (Yazawa et al., 2007; Pitt et al., 2010; Venturi et al., 2013; Kasuya et al., 2016; Yang et al., 2016; Ou et al., 2017; Su et al., 2017; Wang et al., 2019). This is probably because the reconstitution of purified TRICs into bilayers gives rise to frequent brief currents of many varied amplitudes making conventional single-channel characterization difficult. In our hands, the fluctuating amplitudes and rapid gating behavior of many of the purified TRIC currents were similar to the subconductance openings of the SR K^+^ channel (Miller, 1978; Coronado et al., 1980) when examined under identical experimental conditions and led to the idea that TRICs could be SR K^+^ channels (Pitt et al., 2010; Venturi et al., 2013). In view of the irregular TRIC channel behavior, we have now investigated if TRICs are permeable to anions by examining multichannel currents rather than single-channel currents. This allows us to measure the E_rev_ of the bilayer, a measurement that is problematic at the single-channel level if the channel opens briefly to many subconductance levels. Rather than using purified preparations of TRIC proteins, we incorporated membrane vesicles from HEK293 cells expressing RyR2-only or coexpressing RyR2+TRIC-A or RyR2+TRIC-B into planar phospholipid bilayers. In this way, use of harsh purification procedures and detergents which can disrupt ion-channel function is avoided. Moreover, since there is evidence that TRIC proteins can interact directly with RyR to affect RyR channel function, it is possible that RyR may also affect TRIC channel function and that the presence of RyR channels in the bilayer is necessary for stable TRIC channel behavior. Our results provide evidence that TRIC-A and TRIC-B may act as differential regulators of RyR2 gating. We also suggest that the TRIC channels are not ideally selective for monovalent cations as previously thought but may be permeable to both anions and cations.

## Materials and methods

### Expression of TRIC channels in RyR2-expressing HEK293 cells

HEK293 cells stably and inducibly expressing wild-type mouse RyR2 were generated using the Flp-In T-REx system (Life Technologies; Uehara et al., 2017), and mouse TRIC-A or TRIC-B channels were transiently expressed using baculovirus infection (Zhou et al., 2020). Briefly, HEK293 cells expressing RyR2 were cultured in 150-mm dishes. At 70–80% confluency, RyR2 expression was started with 2 µg/ml doxycycline. P2 baculovirus solution for TRIC-A or TRIC-B was added to the culture media (2 ml per 20 ml media) at the time of induction. A single microsomal membrane preparation was obtained from 10 dishes (150 mm) containing either (1) RyR2-only cells, (2) RyR2+TRIC-A cells, or (3) RyR2+TRIC-B cells. Cells were harvested 24 h after induction, rinsed twice with phosphate-buffered saline (PBS), quickly frozen in liquid N_2_, and stored at −80°C before use.

### Isolation of microsomal membrane vesicles from HEK293 cells and SR membrane vesicles from mouse skeletal muscle

HEK293 cells were resuspended in ice-cold hypotonic lysis buffer (1 mM EDTA, 10 mM HEPES, pH 7.4, plus protease inhibitor cocktail, 1 mM DTT, and 5 mM NaF) before homogenization on ice with 10 strokes in a tight fitting glass Douce homogenizer, followed by 15 strokes after the addition of an equal volume of restoration buffer (500 mM sucrose, 10 mM HEPES-Tris, pH 7.2, plus protease inhibitor cocktail, 1 mM DTT, and 5 mM NaF) and centrifugation at 6,000 × *g* for 15 min at 4°C. Microsomes were collected by centrifugation of the supernatant at 100,000 × *g* for 45 min at 4°C. The pellet was resuspended in a buffer containing 250 mM sucrose and 10 mM HEPES pH 7.2 + 5 mM NaF. The membrane vesicles were snap-frozen in aliquots in liquid N_2_ and stored at −80°C. The light SR membrane fraction, which is rich in SR K^+^ channels, was isolated from mouse skeletal muscle as previously described (Venturi et al., 2013).

### Production of recombinant TRIC-B in the wheat germ system

Recombinant TRIC-B proteins were produced using the wheat germ cell-free system according to the manufacturer’s instructions (ProteoLiposome BD Expression Kit; CellFree Sciences; Goren et al., 2009). Murine TRIC-B mRNA was prepared by in vitro transcription using pEU-TRIC-B as the template. 10 μg template DNA or no DNA was transcribed (37°C, 6 h). The translation reaction was performed in the presence of liposomes (2 mg/ml final concentration) using the bilayer and dialysis method with a bottom layer of 0.4 ml and an upper layer of 1.6 ml. After the synthesis reaction (15°C, 63 h), the translated protein was mixed with 2 ml of 80% (wt/vol) Accudenz (Accurate Chemical & Scientific) solution (in 10 mM HEPES-NaOH [pH 7.4], 150 mM NaCl, 1 mM DTT, and 1 mM MgCl_2_). The resultant 40% Accudenz solution containing the synthesized protein was placed in the bottom of a centrifuge tube and overlaid with 6.8 ml of 1.2 M sucrose solution, 1.0 ml of 0.3 M sucrose solution (in 10 mM HEPES-NaOH [pH 7.4], 150 mM NaCl, 1 mM DTT, and 1 mM MgCl_2_). The gradient was centrifuged at 189,000 × *g* for 4 h at 4°C in a SW41 (Beckman Coulter). After ultracentrifugation, proteoliposomes were sharply concentrated at the interface between the 1.2 M sucrose solution and the upper 0.3 M sucrose solution owing to their low density. Proteoliposomes were then aliquoted and stored at −80°C for subsequent electrophysiological analysis.

### Expression of recombinant TRIC-B in yeast

TRIC-B proteins were prepared as described in Pitt et al. (2010) but with a solubilizing detergent consisting of a mixture of 1% 3-((3-cholamidopropyl) dimethylammonio)-1-propanesulfonate (CHAPS) and 5 mg/ml phosphatidylcholine (PC; Pitt et al., 2010). Briefly, TRIC-B proteins carrying a His-tag were stably expressed in *Pichia pastoris*, solubilized with CHAPS/PC from total microsomal fractions, and enriched using a Ni-affinity resin. Purified TRIC-B proteins were concentrated, aliquoted, and stored at −80°C until use in electrophysiological measurements.

### Western blotting

**Figure S1.**
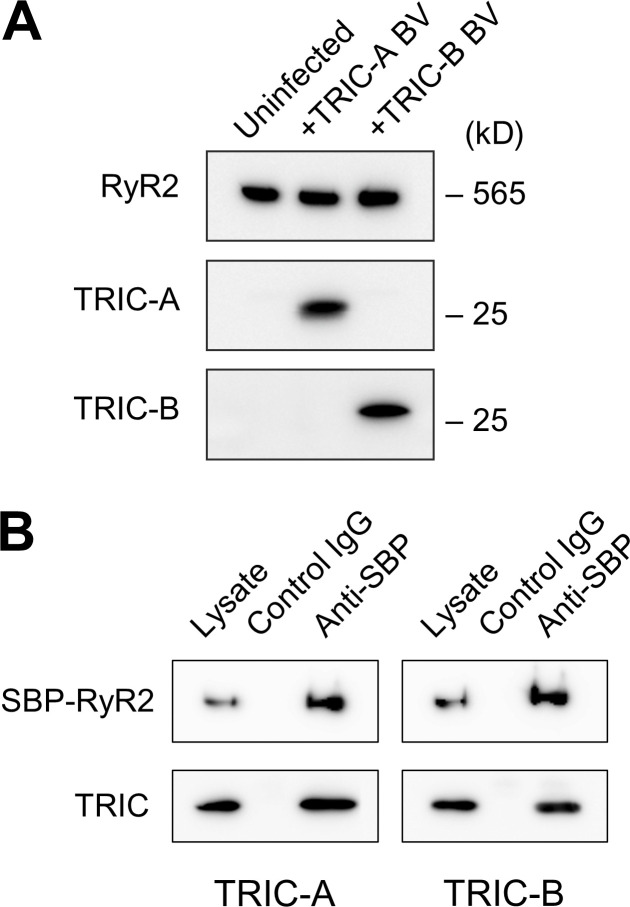
**Expression of TRIC channels and their interaction with RyR2. (A)** Western blots of the microsomal membrane vesicles isolated from RyR2-expressing HEK293 cells that were either uninfected (left), infected with TRIC-A baculovirus (BV; center), or TRIC-B BV (right). **(B)** Interaction of TRIC channels with RyR2. SBP-tagged RyR2-expressing HEK293 cells were infected with TRIC-A (left) or TRIC-B (right) BV. The cell lysate was immunoprecipitated with control IgG or anti-SBP antibody. Lysate and the immunoprecipitated product were analyzed by Western blotting. Note that both TRIC-A and TRIC-B were coimmunoprecipitated with RyR2. Source data are available for this figure: SourceData FS1.

Microsomal proteins were separated on SDS-PAGE (sodium dodecyl sulfate polyacrylamide gel electrophoresis) using a 3–15% linear gradient gel and transferred onto a PVDF (polyvinylidene fluoride) membrane. The membrane was probed with primary antibodies against RyR (Chugun et al., 2003), anti-TRIC-A, and TRIC-B (Yazawa et al., 2007), followed by horseradish peroxidase-labeled goat anti-rabbit IgG secondary antibody (SeraCare Life Sciences, Inc.). A positive band was detected by chemiluminescence using ImmunoStar LD (Fujifilm Wako) as a substrate. Fig. S1 A shows Western blots of the microsomal membrane vesicles isolated from RyR2-expressing HEK293 cells that were either uninfected or infected with TRIC-A or TRIC-B baculovirus.

### Coimmunoprecipitation

Coimmunoprecipitation experiments between RyR2 and TRIC channels were performed by using a streptavidin-binding peptide (SBP)-tagged RyR2, in which the SBP-tag was inserted in the divergent 2 region (after Arg1358 of mouse RyR2). This is because there are not many anti-RyR2 antibodies suitable for immunoprecipitation (IP) experiments. HEK293 cells stably expressing SBP-RyR2 were infected with either TRIC-A or TRIC-B baculovirus. After 24 h, cells were harvested, rinsed with PBS, and lysed with an IP buffer (0.15 M NaCl, 20 mM MOPSO, pH 7.0, 0.015% Tween-20, and a protease inhibitor cocktail) supplemented with 0.5% CHAPS. After centrifugation, the supernatant was diluted with four volumes of IP buffer to reduce the CHAPS concentration to 0.1% and incubated with Dynabeads protein G (Invitrogen) and anti-SBP monoclonal antibody (SB19-C4; Santa Cruz Biotechnology) or control IgG for 2 h at room temperature. After extensive washing of the beads with IP buffer, the bound protein was eluted with SDS sample buffer, and Western blotting was performed to detect the co-immunoprecipitated TRIC-A and TRIC-B with RyR2 as described above.

### Single-channel experiments and analysis

Membrane vesicles were incorporated into planar phosphatidylethanolamine lipid bilayers as previously described (Sitsapesan and Williams, 1994b; Venturi et al., 2013), and K^+^ channel current fluctuations were recorded under voltage-clamp conditions in symmetrical 210 mM KPIPES or 210 mM KCl solutions with 2 µM free Ca^2+^, pH 7.2 (perfusion solutions). The *trans* chamber was held at the ground and the *cis* chamber was clamped at various potentials to the ground. SR vesicles incorporate into bilayers in a fixed orientation (Sitsapesan and Williams, 1994b) such that the *cis* chamber corresponds to the cytosolic face of the SR channels and the *trans* chamber to the luminal side. Experiments were performed at 22 ± 2°C.

**Figure S2.**
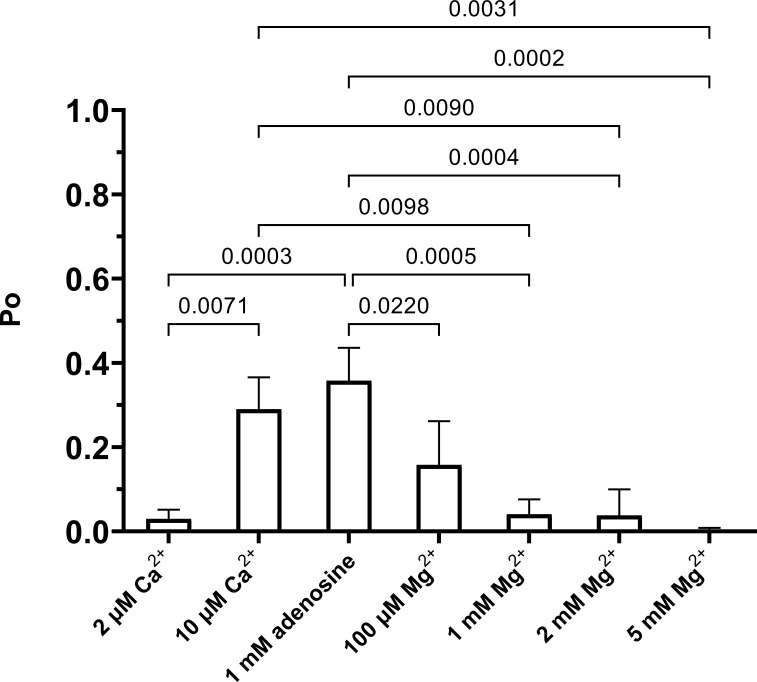
**Effect of adenosine and Mg**^**2+**^ **on the activated RyR2 channel from RyR2-expressing HEK293 cells.** The effect of adenosine and following addition of Mg^2+^ on Po for RyR2 derived from HEK293 cells expressing RyR2-only. The traces were recorded in symmetrical 210 mM KPIPES with 2 µM free Ca^2+^, pH 7.2, at the holding potential of −30 mV, with treatments shown in the figure. One-way ANOVA gives significant differences with P < 0.0001 (*n* = 3). Adjusted P values (Tukey’s correction) used for multiple comparisons are labeled above each pair of comparison if there is significant difference. Values are of the mean ± SEM.

The free [Ca^2+^] and pH of the perfusion solutions were measured simultaneously using a Ca^2+^ electrode (Orion 93-20; Thermo Fisher Scientific) and a Ross-type pH electrode (Orion 81-55; Thermo Fisher Scientific). The perfusion solutions contained normally ≤2 µM free [Ca^2+^]. If the concentration was lower, CaCl_2_ was added to bring the concentration to 2 µM. To make these measurements, a fresh set of standard solutions, made from the relevant perfusion solution, was made for each measurement of free [Ca^2+^] as described by Bers (1982). Subsequent additions of CaCl_2_ were made to the *cis* chamber to bring the [Ca^2+^] to 10 µM, 100 µM, 1 mM, and 2 mM during the experiment. RyR2 incorporation to bilayers was confirmed by the response of the channels to increasing [Ca^2+^] and to other RyR2 regulators such as adenosine and Mg^2+^ (Fig. S2).

Single-channel recordings were digitized at 20 kHz and recorded on computer hard drive using pClamp (v 11.1; Molecular Devices). Blinding was performed before analysis of the traces by coding the filenames of the traces and hiding the recording conditions, such as [Ca^2+^] or type of cell. The recorded traces were then idealized for the detection of open and closed events using Clampfit (v 11.1; Molecular Devices). Before idealization, traces were filtered at 4 kHz (−3 db) if RyR2 gating was being monitored and 800 Hz if SR K^+^ channels or TRIC channel function was being investigated. Open and closed channel levels were assessed using manually controlled cursors. For Po measurements, the holding potential was switched every 15 s between +30 and −30 mV for 2 min. Po was calculated by 50% threshold analysis (Colquhoun and Sigworth, 1983), and the Po at +30 and −30 mV was taken as the average Po for that potential over the 2-min recording time. Binomial analysis (see supplemental text at the end of the PDF) was used to check estimates of the likely number of channels in the bilayer (Auerbach and Sachs, 1984; Colquhoun and Hawkes, 1995).

### Statistics

Statistical analysis was performed using GraphPad Prism (GraphPad Software) and SPSS (released 2021. IBM SPSS Statistics for Windows, Version 28.0.: IBM Corp). Data were expressed as the mean ± SEM and *n* ≥ 3. Samples represent single bilayer experiments where microsomal membrane vesicles were fused with the bilayer and RyR2 channel(s) were incorporated. The Shapiro–Wilk and Kolmogorov–Smirnov normality tests were performed in the data sets to check the distribution of the data. Statistical analysis of historical data using hierarchical linear mixed models has shown that the best-fit model is equivalent to an ANOVA procedure and that the intraclass correlation is very low. Therefore, a one-way ANOVA (or a Kruskal–Wallis test when data did not follow a normal distribution) was performed to compare the mean difference between three or more groups with one independent variable. To assess if the modulation of RyR2 Po by TRIC is affected differently by cytosolic Ca^2+^ concentration, i.e., to compare the mean difference between groups from two independent variables, we used a linear mixed model with two independent variables: (1) “cytosolic [Ca^2+^]” (with five levels of subsequent increases of [Ca^2+^]: 2, 10, 100 μM, 1 and 2 mM Ca^2+^), and (2) “type of HEK293 cells” (with three levels: [1] RyR2-only, [2] RyR2+TRIC-A, and [3] RyR2+TRIC-B). We tested for interaction effects between the two independent variables and also for the main effects of each of the two independent variables. Following a statistically significant result for interaction effects, we assessed the source of the interaction by testing for simple effects. Since the increases in cytosolic [Ca^2+^] were subsequent, the data in each recording are correlated. To preserve these correlations in the model, we also included a random effects factor that determined the identity of the single-channel recording for each measurement. The random effects factor allowed to use correlated data with missing values in a sequence of [Ca^2+^] increases, such as in the rare cases where a bilayer broke before reaching the final [Ca^2+^]. The model yields identical results to a repeated measures two-way ANOVA when there are no missing values in the data. For all tests where multiple comparisons were performed, we used Dunn’s correction for Kruskal–Wallis test or Tukey’s correction for one-way ANOVA or Sidak correction for the linear mixed model, as indicated in the figure legends. A P value <0.05 was considered statistically significant and the details are described in each figure legend.

### Materials

All chemicals were purchased from VWR or Sigma-Aldrich unless otherwise stated. All solutions were prepared in deionized water and filtered through a 0.45-µM pore diameter filter (Millipore).

### Online supplemental material

**Figure S3.**
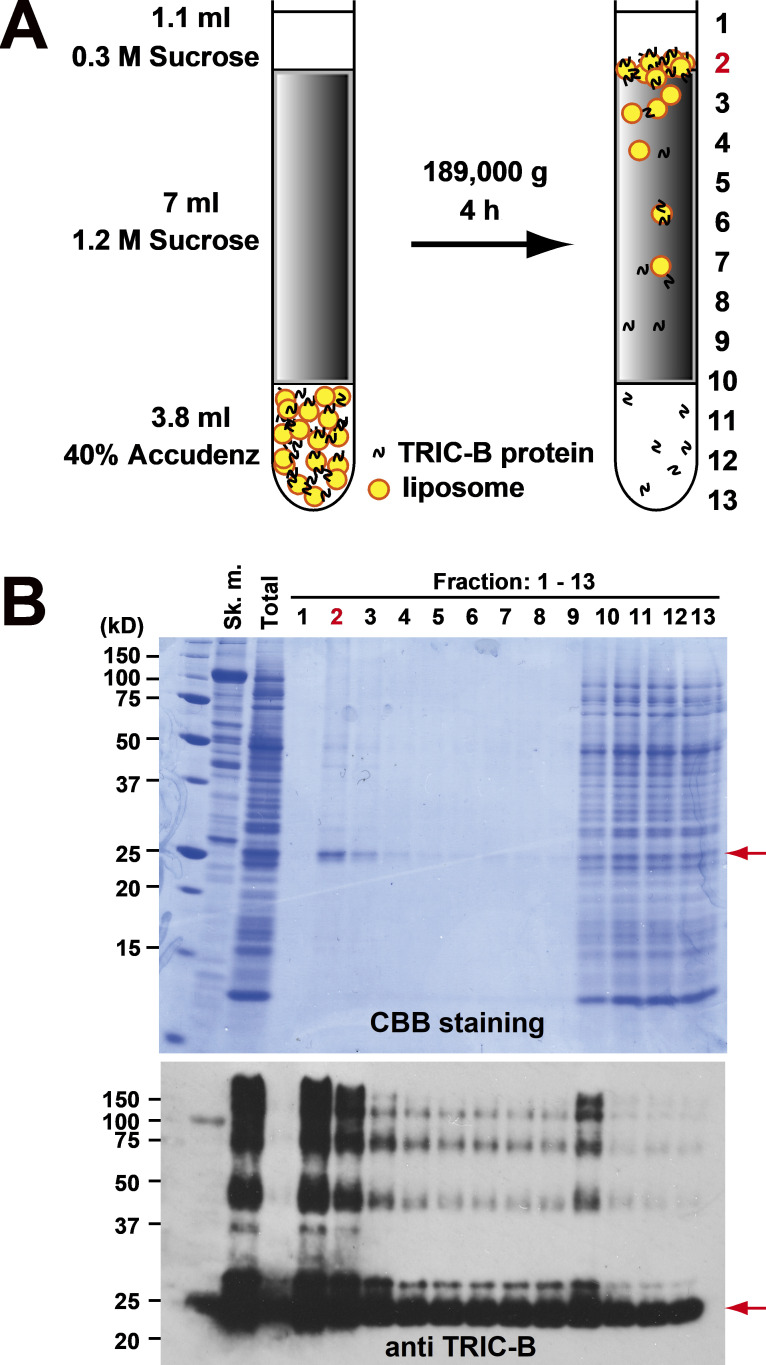
**Purification of TRIC-B proteins by density gradient ultracentrifugation. (A)** Schematic representation of the proteoliposomes containing TRIC-B proteins by wheat-germ translation system. **(B)** SDS-PAGE analysis of proteoliposomes. After the synthesis reaction, the reaction mixture was subjected to a density gradient and fractions were collected from the top of the tube. Synthesized proteins in each fraction were applied to SDS-PAGE and stained CBB (upper panel) or transferred PVDF and probed using TRIC-B antibody (lower panel). Sk. m., mouse skeletal muscle protein.

Fig. S1 A shows Western blot results of microsomal membrane vesicles of coexpressed mouse TRIC-A or TRIC-B with mouse RyR2 in HEK293 cells, and Fig. S1 B shows coimmunoprecipitation results of SBP-tagged RyR2 and TRIC-A or TRIC-B. Fig. S2 shows the effects of adenosine and Mg^2+^ on the activated RyR2 channel from RyR2-expressing HEK293 cells. Fig. S3 shows the purification method of recombinant TRIC-B protein. Tables S1, S2, S3, and S4 list adjusted P values for simple-effect comparison of the effect of co-expression of TRIC-A or TRIC-B on the Po of RyR2 under different cytosolic [Ca^2+^] at different holding potentials, −30 mV (Tables S1 and S2) or +30 mV (Tables S3 and S4). Methods for estimation of the number of channels are provided in the supplemental text at the end of the PDF.

## Results

### TRIC modulation of RyR2 single-channel behavior

To examine if TRIC channels can directly influence the single-channel behavior of RyR2, we expressed TRIC-A or TRIC-B in HEK293 cells over-expressing RyR2 using baculovirus. Expression of RyR2, TRIC-A, and TRIC-B was confirmed by Western blotting (Fig. S1 A). Coimmunoprecipitation experiments with SBP-tagged RyR2 clearly demonstrated a physical interaction between RyR2 and TRIC-A or TRIC-B (Fig. S1 B).

**Figure 1.**
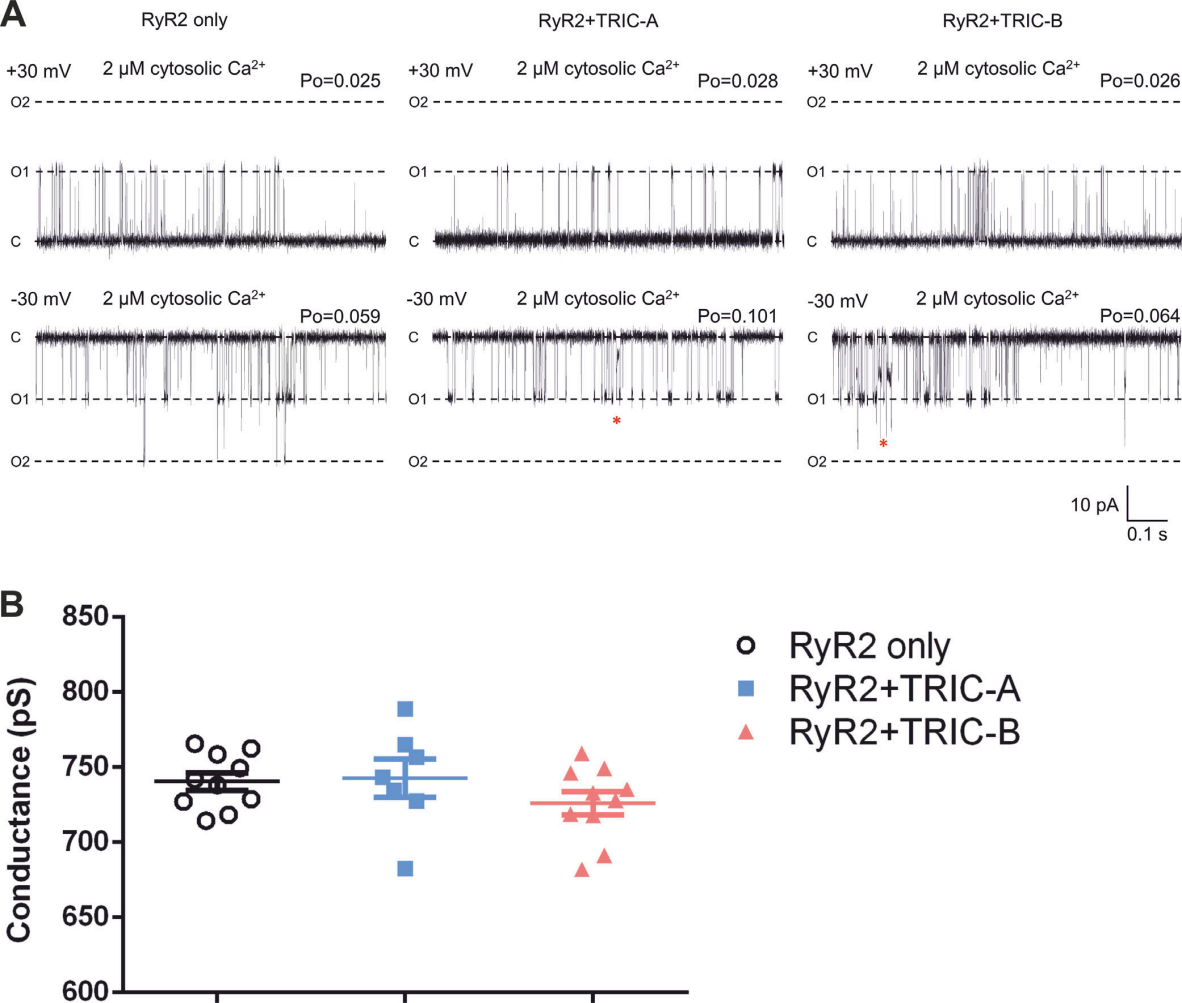
**The effects of coexpressing TRIC-A or TRIC-B with RyR2 on the function of RyR2 channels incorporated into bilayers at 2 μM free Ca**^**2+**^**. (A)** Representative current fluctuations of RyR2 channels from the HEK293 cells expressing RyR2-only (left), RyR2+TRIC-A (middle), and RyR2+TRIC-B (right) at the holding potentials of ±30 mV in symmetrical 210 mM KPIPES, 2 μM free Ca^2+^. The Po (measured over 2 min) is shown above each trace. The zero current level is indicated by C, a single full open channel level by O1, and double full open channel level by O2. The red asterisks highlight subconductance events. **(B)** Single-channel K^+^ conductance of RyR2 channels from HEK293 cells expressing RyR2-only (black circles), RyR2+TRIC-A (blue squares), and RyR2+TRIC-B (red triangles) in the same solutions as in A. The amplitudes in I–V curve of conductance were measured from multiple traces at ±30 and 0 mV. One-way ANOVA for the independent variable, “type of HEK293 cells,” is not significant with P = 0.72 (n_RyR2_ = 10, n_RyR2+TRIC-A_ = 7, n_RyR2+TRIC-B_ = 10). Symbols indicate values from individual experiments and bars indicate mean ± SEM.

**Figure 2.**
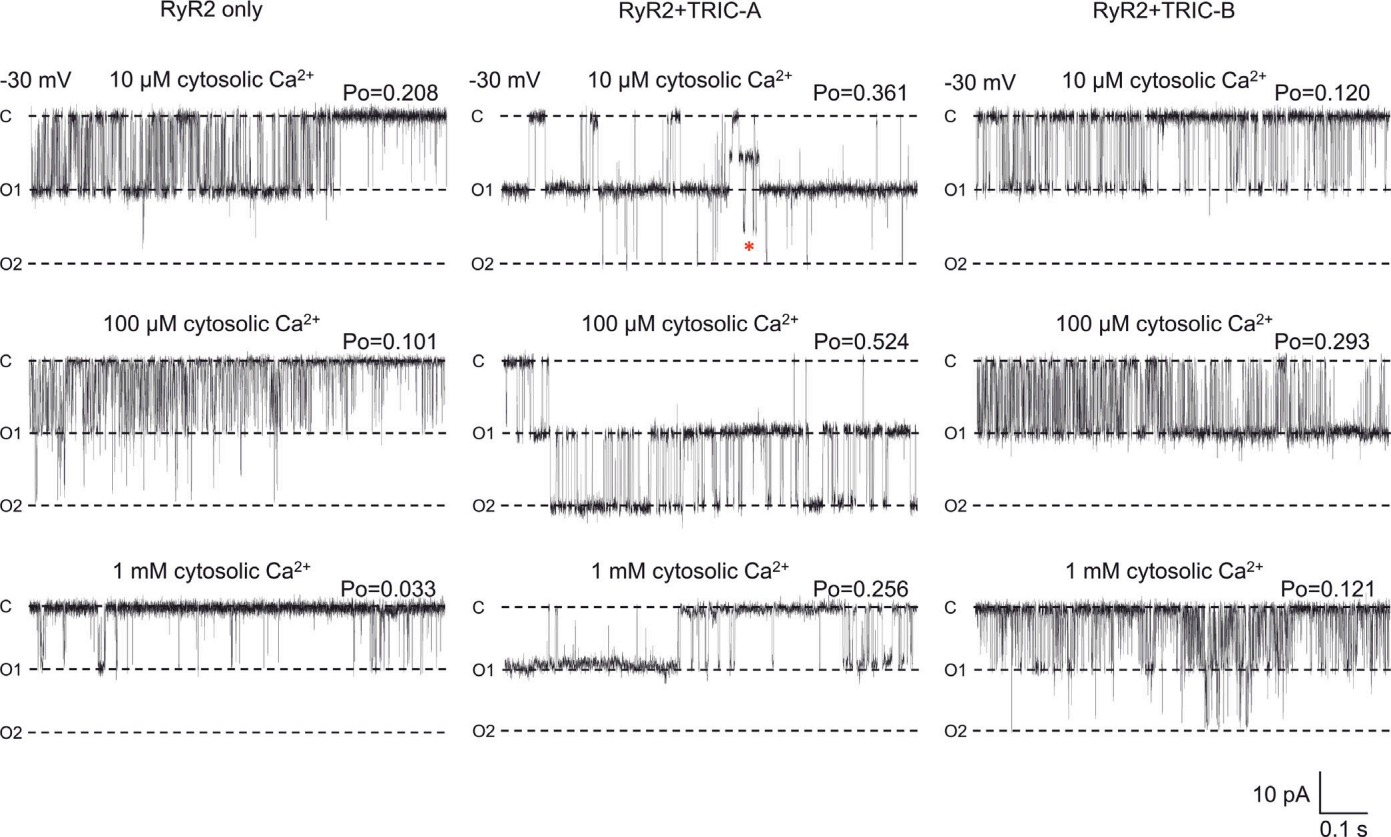
**The effects of coexpressing TRIC-A or TRIC-B with RyR2 on the response of RyR2 channels to increasing cytosolic [Ca**^**2+**^**].** Typical gating behavior of RyR2 is shown from the HEK293 cells expressing RyR2-only (left), RyR2+TRIC-A (middle), and RyR2+TRIC-B (right) at −30 mV in symmetrical 210 mM KPIPES, at the indicated free [Ca^2+^]. In symmetrical 210 mM KPIPES and 10 µM cytosolic Ca^2+^, RyR single-channel conductance from the RyR2-only, RyR2+TRIC-A, and RyR2+TRIC-B cells was 735.7 ± 12.7 pS, 735.0 ± 19.2 pS, and 726.3 ± 9.0 pS, respectively. When cytosolic Ca^2+^ was raised to 1 mM, RyR conductance decreased to 544 ± 22 pS, 549 ± 34 pS, and 557 ± 10 pS for RyR2-only, RyR2+TRIC-A, and RyR2+TRIC-B cells, respectively (n_RyR2_ = 10, n_RyR2+TRIC-A_ = 7, n_RyR2+TRIC-B_ = 10). The Po (measured over 2 min) is shown above each trace. The zero current level is indicated by C and the fully open channel levels by O1 and O2. The red asterisk highlights subconductance events.

**Figure 3.**
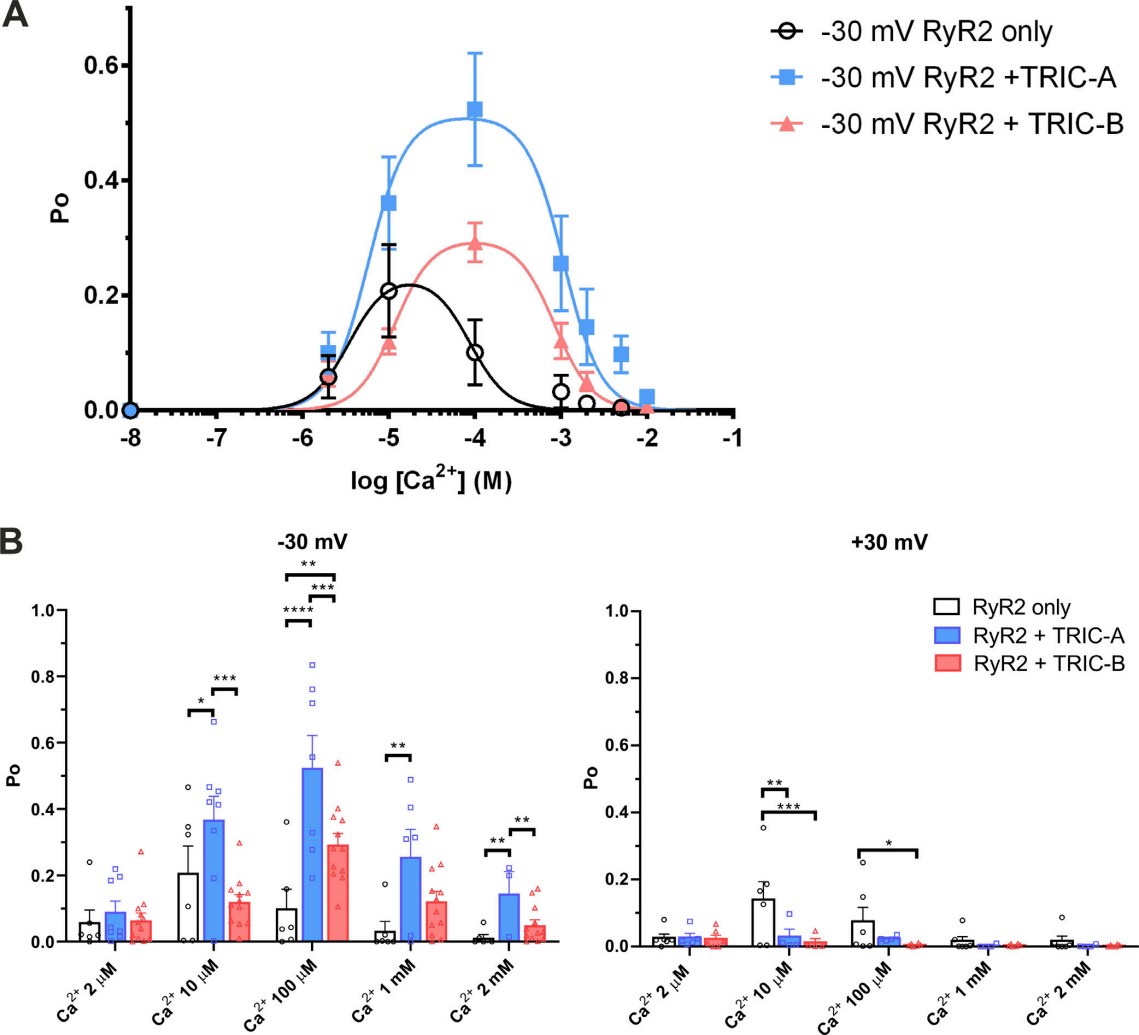
**The effects of co-expressing TRIC-A or TRIC-B with RyR2 on the relationship between Po and cytosolic [Ca**^**2+**^**]. (A)** Relationship between [Ca^2+^] and Po for RyR2 derived from HEK293 cells expressing RyR2-only (black), RyR2+TRIC-A (blue), and RyR2+TRIC-B (red) at −30 mV. Lines display non-linear bell-shaped fitted curves. EC_50_ values are 3.6 µM for RyR2-only, 5.8 µM for RyR2+TRIC-A, and 11.7 µM for RyR2+TRIC-B, and IC_50_ values are 87 µM for RyR2-only, 1.08 mM for RyR2+TRIC-A, and 0.84 mM for RyR2+TRIC-B. **(B)** The effect of holding potential (−30 mV on the left; +30 mV on the right) on Po for RyR2 derived from HEK293 cells expressing RyR2-only (black), RyR2+TRIC-A (blue), and RyR2+TRIC-B (red). For the statistical analysis, we used a linear mixed model with two independent variables: (1) “cytosolic [Ca^2+^]” and (2) “type of HEK293 cells.” To preserve correlations within recordings, a Random Effects factor was included in the model that determined the identity of the single-channel recording for each measurement (as discussed in Materials and methods). For the −30 mV on the left: interaction effect for cytosolic [Ca^2+^] by type of HEK293 cells P = 0.000008; main effect of cytosolic [Ca^2+^], P = 4.794 × 10^−11^; main effect of type of HEK293 cells, P = 0.000025 (n_RyR2_ = 6, n_RyR2+TRIC-A_ = 3–8, n_RyR2+TRIC-B_ = 13). Adjusted P values (Sidak correction) for simple effects are listed in Tables S1 and S2. For the +30 mV on the right: interaction effect for cytosolic [Ca^2+^] by type of HEK293 cells, P = 0.0219; main effect of cytosolic [Ca^2+^], P = 0.0024; main effect of type of HEK293 cells; P = 0.0423 (n_RyR2_ = 6, n_RyR2+TRIC-A_ = 4–5, n_RyR2+TRIC-B_ = 6). Adjusted P values (Sidak correction) for simple effects are listed in Tables S3 and S4. Simple effects tests for type of HEK293 cells comparing (1) RyR2-only, (2) RyR2+TRIC-A, and (3) RyR2+TRIC-B are indicated by asterisks (*: P < 0.05; **: P < 0.01; ***: P < 0.001; ****: P < 0.0001). Values are of the mean ± SEM.

We isolated the mixed membrane fraction from each HEK293 cell type (RyR2-only, RyR2+TRIC-A, or RyR2+TRIC-B). After incorporating vesicles into phospholipid bilayers, we examined the RyR2 function under conditions where K^+^ is the permeant ion (symmetrical 210 mM K^+^, symmetrical 2 μM free Ca^2+^). Fig. 1 A illustrates representative current fluctuations of RyR2 at holding potentials of ±30 mV. The gating behavior of RyR2 appeared similar at this [Ca^2+^], irrespective of coexpression with TRIC-A or TRIC-B, although we did notice what appeared to be a more frequent subconductance state gating. Coexpression with TRIC-A or TRIC-B did not change RyR2 single-channel K^+^ conductance as shown in Fig. 1 B. We investigated if the coexpression of TRIC-A or TRIC-B affected the response of RyR2 to increasing concentrations of cytosolic Ca^2+^, and the resulting typical gating behavior observed at −30 mV is shown in Fig. 2. In virtually all experiments, there were multiple channels gating together in the bilayer and, therefore, we were unable to perform lifetime analysis (which requires the presence of just one channel). Visual inspection of the traces, however, shows the longer openings and higher Po levels that were particularly prevalent in the RyR2+TRIC-A experimental group. Fig. 3 A illustrates the mean Po changes for the three groups over the full range of activating and inactivating cytosolic [Ca^2+^]. Two key points can be made: (1) without coexpression of TRIC channels, the maximum Po achieved with cytosolic Ca^2+^ as the sole activator is low at ∼0.2. The coexpression of TRIC-A is particularly effective at potentiating the effect of cytosolic Ca^2+^, bringing the maximum Po to ∼0.55. (2) Inactivation by high [Ca^2+^] occurs at much lower [Ca^2+^] when RyR2 is expressed without TRIC channels. The half maximal inhibitory concentration (IC_50_) for RyR2-only is 87 μM, whereas for RyR2+TRIC-A it is 1.08 mM and for RyR2+TRIC-B it is 0.84 mM. For the RyR2-only channels, we would expect a low maximum Po reflecting the fact that Ca^2+^, as a sole activator, is a partial agonist for RyR2 derived directly from cardiac tissue without purification (native channels; Ashley and Williams, 1990; Sitsapesan and Williams, 1994a; Loaiza et al., 2013; Uehara et al., 2017). Similarly, considering RyR2 inactivation by cytosolic Ca^2+^, our results are in line with most of the published literature where RyR2 inhibition occurs at [Ca^2+^] of 1 mM or lower (Fruen et al., 1994, 1996; Liu et al., 1998; Chamberlain et al., 1984; Meissner and Henderson, 1987; Zimányi and Pessah, 1991; Chu et al., 1993; Copello et al., 1997; Uehara et al., 2017; Wilson et al., 2021). Some groups find dissimilar results on maximum Po or RyR2 inactivation and these can be attributed to different methods of enrichment of RyR2, particularly where detergents have been used for purification (Li and Chen, 2001; Xiao et al., 2007; Meli et al., 2011; Mukherjee et al., 2012). These differences could be attributed to factors such as loss of a closely associated protein, stripping of a lipid, alteration to a ligand binding site by the use of Cs^+^ (Laver et al., 1995), or using oxidized glutathione (Gomez and Yamaguchi, 2014).

**Figure 4.**
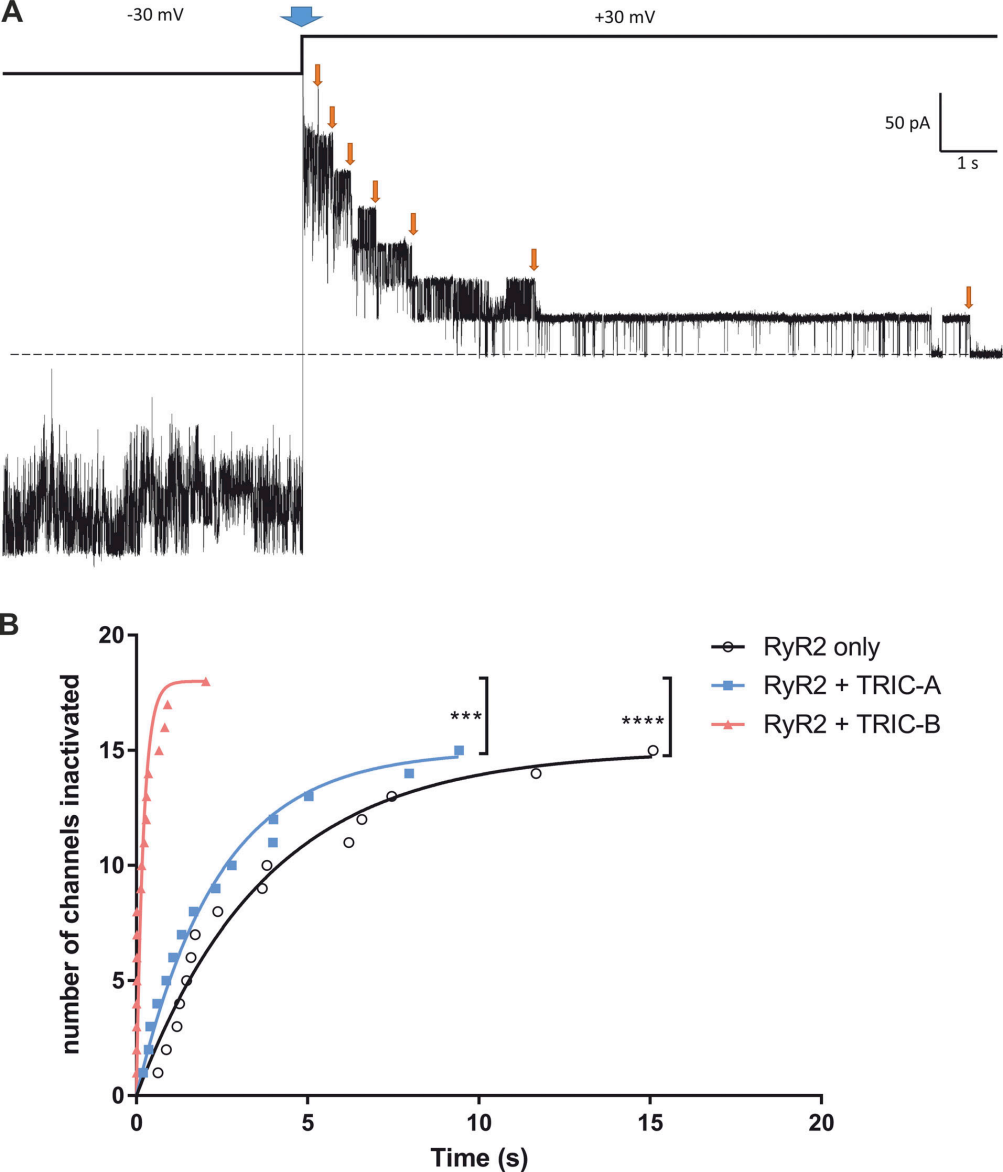
**Voltage-dependent inactivation of RyR2. (A)** Example of voltage-dependent inactivation of RyR2 channels that results from switching the holding potential from −30 to +30 mV in a bilayer where multiple RyR2 channels were gated prior to the voltage change. The blue arrow indicates the time of the voltage change and the orange arrows indicate the times of individual channel inactivation. The dashed line indicates 0 pA. **(B)** Graph comparing the times to channel inactivation after changing the holding potential from −30 to +30 mV in the RyR2 derived from HEK293 cells expressing RyR2-only (black), RyR2+TRIC-A (blue), and RyR2+TRIC-B (red). The curves were fitted according to the equation: $y=n\left(1-e^{-kt}\right)$, where *t* = time in seconds, *n* is the number of channels (n_RyR2_ = 15, n_RyR2+TRIC-A_ = 15, n_RyR2+TRIC-B_ = 18), and k is the rate constant (k_RyR2_ = 0.264, k_RyR2+TRIC-A_ = 0.422, k_RyR2+TRIC-B_ = 4.89). The time for half the channels to become inactivated (half decay time, t_1/2_) was 2.62, 1.64, and 0.14 s for RyR2-only, RyR2+TRIC-A, and RyR2+TRIC-B, respectively. Kruskal–Wallis test: P = 0.000003. Post-hoc multiple comparison with Dunn’s correction: significant difference between the t_1/2_ of RyR2 derived from HEK293 cells expressing RyR2+TRIC-B and the t_1/2_ of cells expressing RyR2-only (indicated by **** for P = 0.000007) or RyR2+TRIC-A (indicated by *** for P = 0.000483).

The data shown in Fig. 3 A reflect only the relationships between cytosolic [Ca^2+^] and Po at the holding potential of −30 mV. However, RyR2 is known to undergo voltage-dependent inactivation in a manner that is dependent on the activating ligands (Sitsapesan et al., 1995; Hill and Sitsapesan, 2002) and we observed this in the current study. After switching to +30 mV, we observed inactivation, and this markedly affected the measurement of Po as shown in Fig. 3 B. The voltage-dependent inactivation did not significantly affect the overall Po measurements of the RyR2-only channels, but it can be seen that in the experiments where TRIC channels were coexpressed, the average steady-state Po was reduced to near zero. Fig. 4 A illustrates a typical example of voltage-dependent inactivation in an experiment in which there were at least seven channels in the bilayer. The channels were gating stochastically in steady state at −30 mV, but when the voltage was switched to +30 mV (at the blue arrow), one by one (orange arrows), the channels shut down and did not reopen until the potential was reversed to −30 mV. Only 54% (15 of 28 channels) of RyR2 channels derived from HEK293 cells expressing only RyR2 inactivated following the voltage-switch in comparison to 79% (15 of 19 channels) of channels that were co-expressed with TRIC-A and 69% (18 of 26 channels) of channels that were co-expressed with TRIC-B. Fig. 4 B illustrates a cumulative plot of the times to inactivation following the switch to +30 mV yielding the times for half the channels to be inactivated (t_1/2_) of 2.62 s for RyR2-only, 1.64 s for RyR2+TRIC-A, and 0.14 s for RyR2+TRIC-B. Thus, inactivation was particularly fast when TRIC-B was co-expressed with RyR2 being significantly faster than for RyR2-only (P = 0.000007) and RyR2+TRIC-A (P = 0.000483) channels.

**Figure 5.**
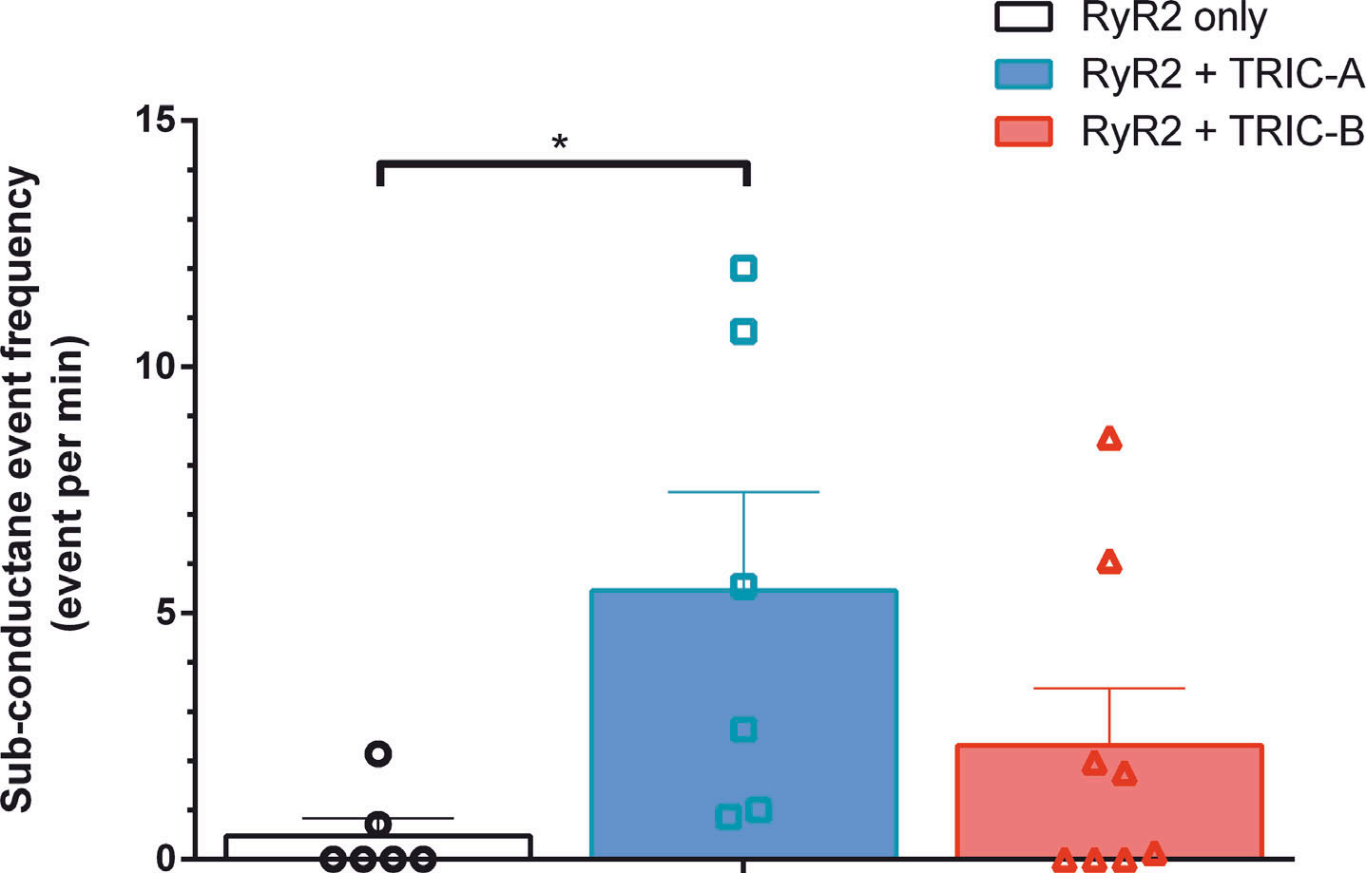
**The effects of co-expressing TRIC-A or TRIC-B with RyR2 on the frequency of subconductance events.** Bar chart showing the frequency of subconductance open events >2 ms in duration at 10 µM cytosolic free [Ca^2+^] at −30 mV, in the same solutions as in Fig. 2, for RyR2 derived from HEK293 cells expressing RyR2-only (black), RyR2+TRIC-A (blue), and RyR2+TRIC-B (red). The Kruskal–Wallis test for one independent variable, type of cell, P = 0.0271. Post-hoc multiple comparison with Dunn’s correction: significant difference between the event frequency of RyR2 derived from HEK293 cells expressing RyR2-only or cells expressing RyR2+TRIC-A is indicated by * for P = 0.0287 (n_RyR2_ = 6, n_RyR2+TRIC-A_ = 6, n_RyR2+TRIC-B_ = 8). Symbols indicate values from individual experiments and bars show mean ± SEM.

On examination of the single-channel traces, we observed occasional gating into sub-conductance states. Examples of these can be seen in Fig. 1 A at −30 mV and 2 μM cytosolic Ca^2+^ in the RyR2+TRIC-A and RyR2+TRIC-B traces and in Fig. 2 B at −30 mV and 10 μM Ca^2+^ in the RyR2+TRIC-A trace. RyR2 single-channel events are very brief and the duration of the majority of events is close to the minimum resolvable duration. We therefore investigated if the coexpression of TRIC channels was associated with an increased frequency of subconductance states by counting only those subconductance states that were ≥2 ms to avoid misclassifying rapid gating as subconductance events. We only monitored RyR2 substate events at 10 μM cytosolic Ca^2+^ because substate detection was inaccurate when Po was high and there were multiple channels in the bilayer, and below 10 μM cytosolic Ca^2+^, we did not observe many subconductance events. Fig. 5 shows that subconductance events were rare in the RyR2-only channel group (on average <1 event per min). Although there was a trend toward more subconductance events when either TRIC-A or TRIC-B was coexpressed, this was only statistically significant when TRIC-A was coexpressed.

### TRIC-channel characteristics

**Figure 6.**
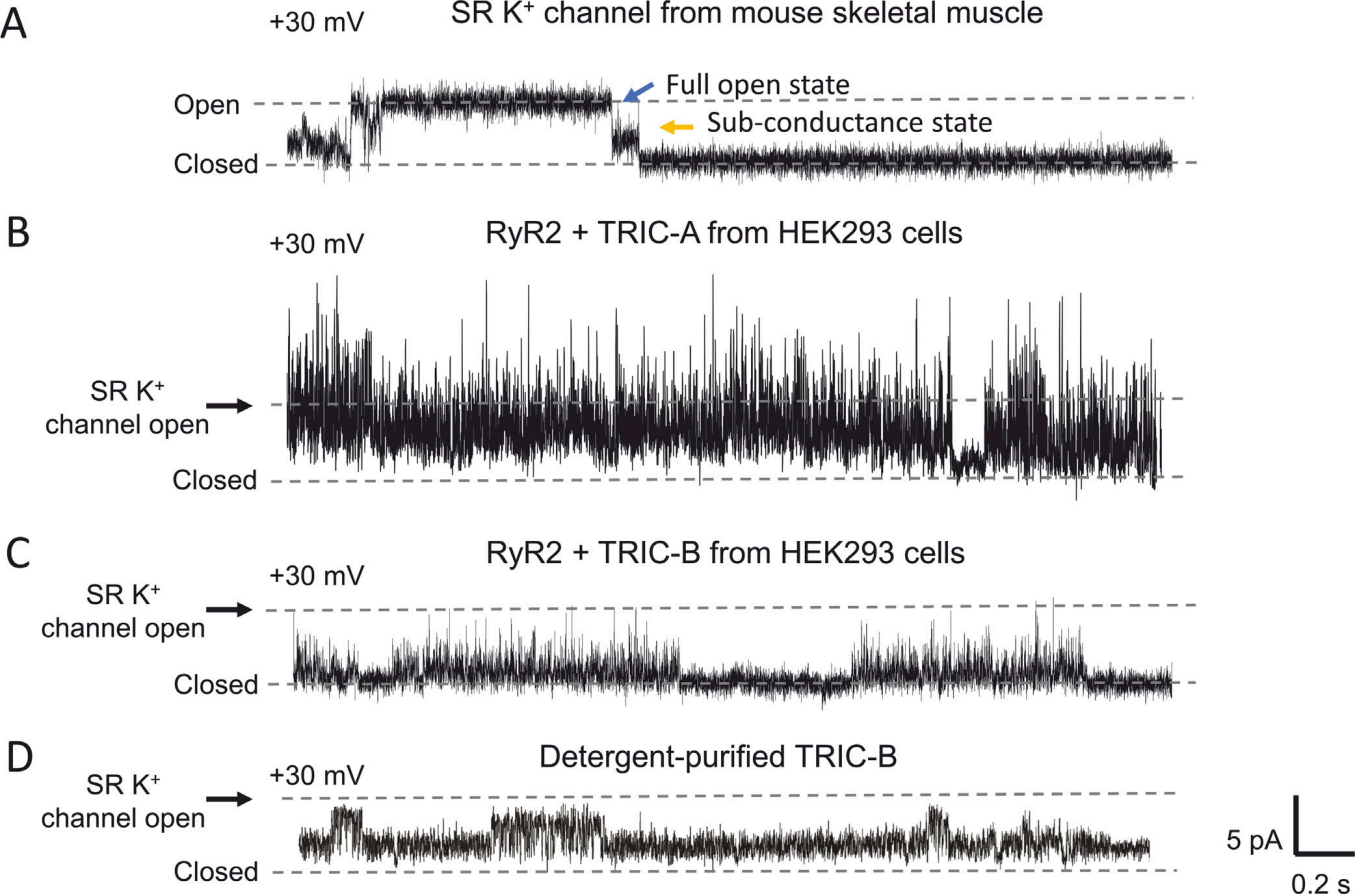
**Comparison of mouse skeletal SR K**^**+**^ **single-channel events with those derived from HEK293 cell vesicles coexpressing TRIC-A or TRIC-B with RyR2 and with detergent-purified TRIC-B. (A)** Typical SR K^+^ channel openings were observed after incorporating mouse skeletal muscle SR vesicles into bilayers in symmetrical 210 mM KPIPES, 2 µM free Ca^2+^ at the holding potential of +30 mV. The zero current level is indicated by “closed” and the fully open channel level by “open.” The orange and blue arrows indicate the characteristic rapidly gating subconductance states and slower gating full conductance openings. So that direct comparison can be made with the TRIC channel currents under identical recording conditions, B–D indicate the amplitude of a full SR K^+^ channel opening with a black arrow and the zero current level with “closed.” **(B)** Example of the currents observed after incorporating vesicles from HEK293 cells coexpressing RyR2+TRIC-A under the same experimental conditions as in A. **(C)** Example of the currents observed after incorporating vesicles from HEK293 cells coexpressing RyR2+TRIC-B under the same experimental conditions as in A. **(D)** Example of current fluctuations obtained after bilayer incorporation of CHAPS-purified TRIC-B channels expressed in yeast under the same experimental conditions as in A.

The rapid gating and varying subconductance states of purified TRIC channels bear some similarities with the properties of the SR K^+^ channels first reported by Miller (Miller, 1978; Labarca and Miller, 1981) supporting the idea that TRIC-A and/or TRIC-B could be the SR K^+^ channels (Pitt et al., 2010; Venturi et al., 2013). To date, however, no publication has shown the exact single-channel characteristics of an SR K^+^ channel in a purified preparation of TRIC protein, and so we investigated if any single-channel behavior that resembled SR K^+^ channel gating could be found in our preparations. Fig. 6 A shows an example of a single-channel recording from an SR K^+^ channel derived from mouse skeletal muscle, gating at +30 mV in symmetrical solutions of 210 mM KPIPES and free [Ca^2+^] 2 μM, pH 7.2. Typical gating behavior is shown where the SR K^+^ channel first enters a subconductance open state before entering the fully open state and then exits the fully open state via a subconductance state. In a total of 34 bilayer experiments where we fused vesicles from HEK cells overexpressing RyR2-only, we did not observe any SR K^+^ channel-like events. We also did not observe any SR K^+^ channel-like activity in a total of 36 bilayer experiments using the RyR2+TRIC-A vesicles and 46 bilayer experiments using the RyR2+TRIC-B vesicles. Fig. 6, B and C, show typical examples of current fluctuations arising from the fusion of vesicles from the RyR2+TRIC-A cells or from the RyR2+TRIC-B cells, respectively, under identical experimental conditions to those shown in panel A. For ease of comparison, the level of an SR K^+^ channel full open event is shown by an arrow to the left of the recording. In panels B and C, it should be noted that there are many rapid gating fluctuations that could be mistaken for SR K^+^ subconductance states but no clear events that resembled full openings.

In panel D, recordings of CHAPS-purified TRIC-B, are shown and, again, there were no single-channel events resembling those of the SR K^+^ channel.

It should be obvious from the recordings in Fig. 6, B–D, that it is impossible to obtain an E_rev_ of a single channel of TRIC-A or TRIC-B because of the rapid gating events and the fact that there is no obvious full open state. Therefore, the most accurate method of finding an E_rev_ for TRIC-A and TRIC-B is to incorporate as many vesicles as possible into the bilayer and then apply a voltage ramp. During this experiment, gradient solutions of 740 mM KCl cytosolic:210 mM KCl luminal were used while a voltage ramp from −50 to +50 mV was applied across the bilayer.

**Figure 7.**
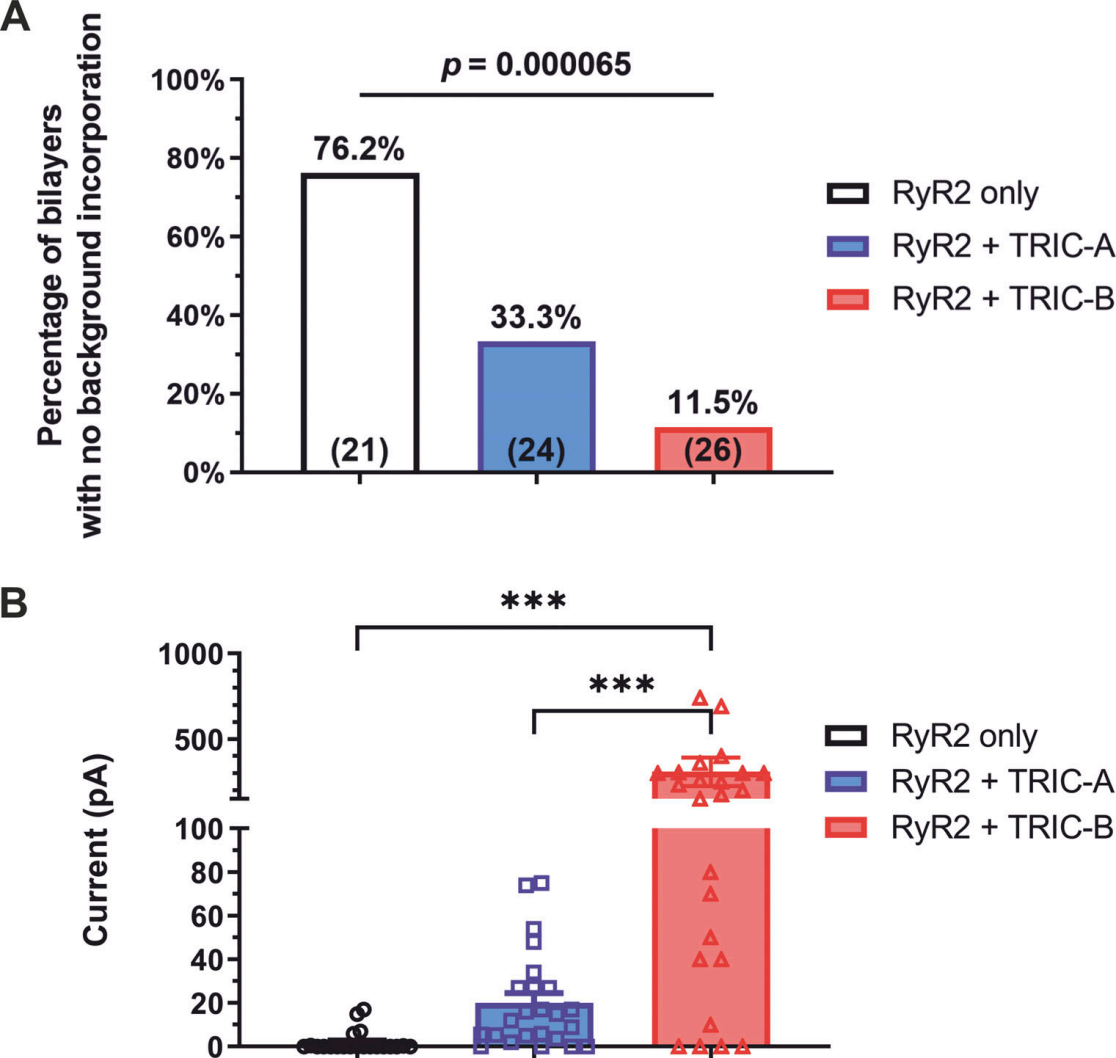
**Effect of co-expressing TRIC-A or TRIC-B on background currents. (A)** The bar chart illustrates the percentage of the bilayer in which no background current was detected after the incorporation of vesicles from HEK293 cells expressing RyR2-only, RyR2+TRIC-A, and RyR2+TRIC-B. Significant differences were detected across the three groups using a χ-square test with a P value of 0.000065. The percentage of bilayers with no background current is indicated in each bar (total number of bilayers: n_RyR2_ = 21, n_RyR2+TRIC-A_ = 24, n_RyR2+TRIC-B_ = 26). **(B)** Mean current amplitudes in KCl gradient conditions after vesicles incorporation are compared for HEK293 cells expressing RyR2-only, RyR2+TRIC-A, and RyR2+TRIC-B. One-way ANOVA: P = 0.000065. Post-hoc multiple comparison with Tukey’s correction: significant differences between RyR2-only and RyR2+TRIC-A vesicle preparations are indicated by *** for P = 0.0003 and between the RyR2+TRIC-A and RyR2+TRIC-B indicated by *** with P = 0.0005 (n_RyR2_ = 21, n_RyR2+TRIC-A_ = 24, n_RyR2+TRIC-B_ = 26). Symbols indicate values from individual experiments and bars indicate mean ± SEM.

Fusion of vesicles from HEK cells expressing only RyR2 led primarily to the incorporation of RyR2 channels although, occasionally, other current fluctuations were also observed. This was expected as HEK cells do have some endogenous ion channels that can incorporate into the bilayer giving rise to “background” currents. Fig. 7 A shows that with the fusion of vesicles from HEK cells expressing only RyR2, 76% of bilayers exhibited no background currents. However, fusion of vesicles from HEK cells coexpressing RyR2 plus TRIC-A or TRIC-B led to increased incorporation of rapidly gating current fluctuations of variable amplitudes along with RyR2. With the RyR2+TRIC-A cells, only 35% of bilayers contained no background currents and with the RyR2+TRIC-B cells, only 11.5% of bilayers contained no background current. These differences were significant and suggest that the extra currents observed in the RyR2+TRIC-A and RyR2+TRIC-B cells were due to TRIC channels.

**Figure 8.**
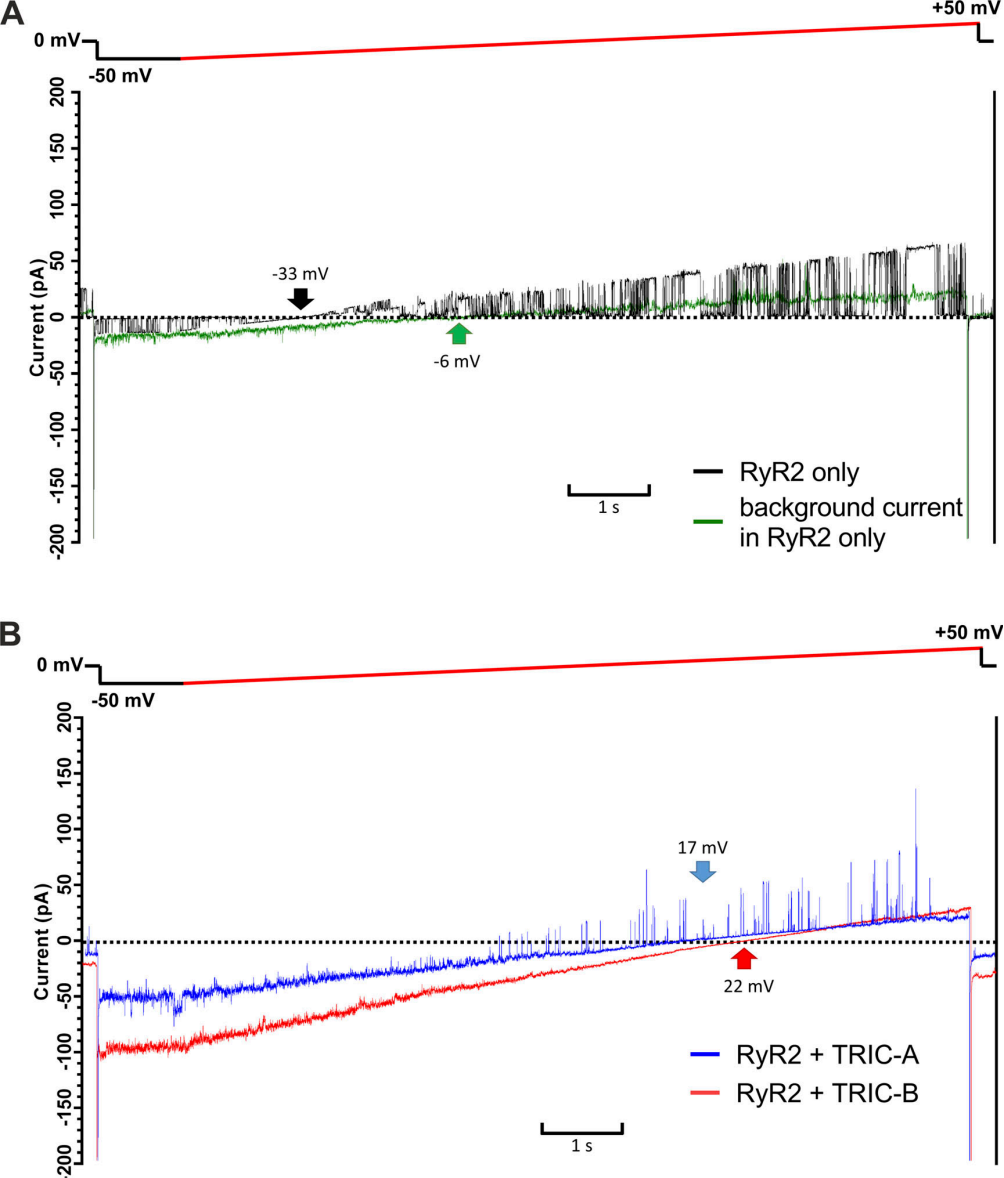
**Effects of coexpressing TRIC-A or TRIC-B with RyR2 on the currents elicited by voltage ramps. (A)** Representative current fluctuations from individual experiments after incorporating vesicles from HEK293 cells expressing only RyR2 in which a single RyR2 channel was incorporated (black) or when a background current was detected (green). The black (RyR2-only) and green (background current) arrows indicate the E_rev_ for the individual experiment. **(B)** Representative currents from individual experiments after incorporating vesicles from HEK293 cells coexpressing RyR2+TRIC-A (blue) or coexpressing RyR2+TRIC-B (red) into the bilayer. The blue (RyR2+TRIC-A) and red (RyR2+TRIC-B) arrows indicate the E_rev_ for the corresponding experiment. Vesicles were incubated with the bilayer for 5–10 min in 740 mM cytosolic:210 mM luminal KCl gradient to allow multiple fusion events (evidenced by step changes in conductance) to occur. Vesicle fusion was halted by perfusing the cytosolic chamber with 210 mM KCl. The 740 mM cytosolic:210 mM luminal KCl gradient was then reapplied. The bilayers were held at 0 mV and then switched to −50 mV for 1 s before applying the voltage ramp (red) from −50 to +50 mV.

Fig. 7 B demonstrates that at 0 mV, the average amplitude of the background currents after vesicle incorporation was very small for the RyR2-only vesicles, in comparison with the background currents arising from RyR2+TRIC-A and RyR2+TRIC-B vesicles. We investigated the E_rev_ values of these background currents by using the voltage ramp protocol as shown in Fig. 8. Fig. 8 A shows what can happen with the RyR2-only cells. When there were RyR2 channels incorporated into the bilayers with no background currents, the E_rev_ was always around −32 mV (see black trace), the calculated E_rev_ for K^+^ in the 740 mM KCl cytosolic:210 mM KCl luminal solutions, as RyR2 is ideally selective for cations (Lindsay et al., 1991). When background currents were incorporated, these were usually small, but we deliberately show an example of an experiment with the highest amount of background current observed for the RyR2-only cells (see green trace) to clearly visualize the ramp current. The E_rev_s of the background currents varied but these were usually more positive than −32 mV indicating that there are endogenous ion channels in HEK cells that exhibit at least some permeability to Cl^−^. Sometimes, both RyR2 and background currents incorporated into the bilayers giving rise to variable E_rev_ values that were more positive than −32 mV.

In contrast, the E_rev_ we obtained from the RyR2+TRIC-A and RYR2+TRIC-B cells was markedly different. Fig. 8 B shows what can happen with the RyR2+TRIC-A and RYR2+TRIC-B cells. Typical examples of ramp currents are shown with the E_rev_ for the ramp indicated with a blue arrow (RyR2+TRIC-A) or a red arrow (RyR2+TRIC-B). Notice that the overall background currents are large and that RyR2 can also be incorporated, thus giving rise to a large degree of variability in the E_rev_. The most important feature to be aware of, however, is that the E_rev_ values for both the RyR2+TRIC-A and the RyR2+TRIC-B cells are more positive than −32 mV, the calculated E_rev_ for K^+^ in the 740 mM KCl cytosolic:210 mM KCl luminal solutions. Thus, the TRIC channels that have been incorporated must be passing some Cl^−^ together with K^+^.

**Figure 9.**
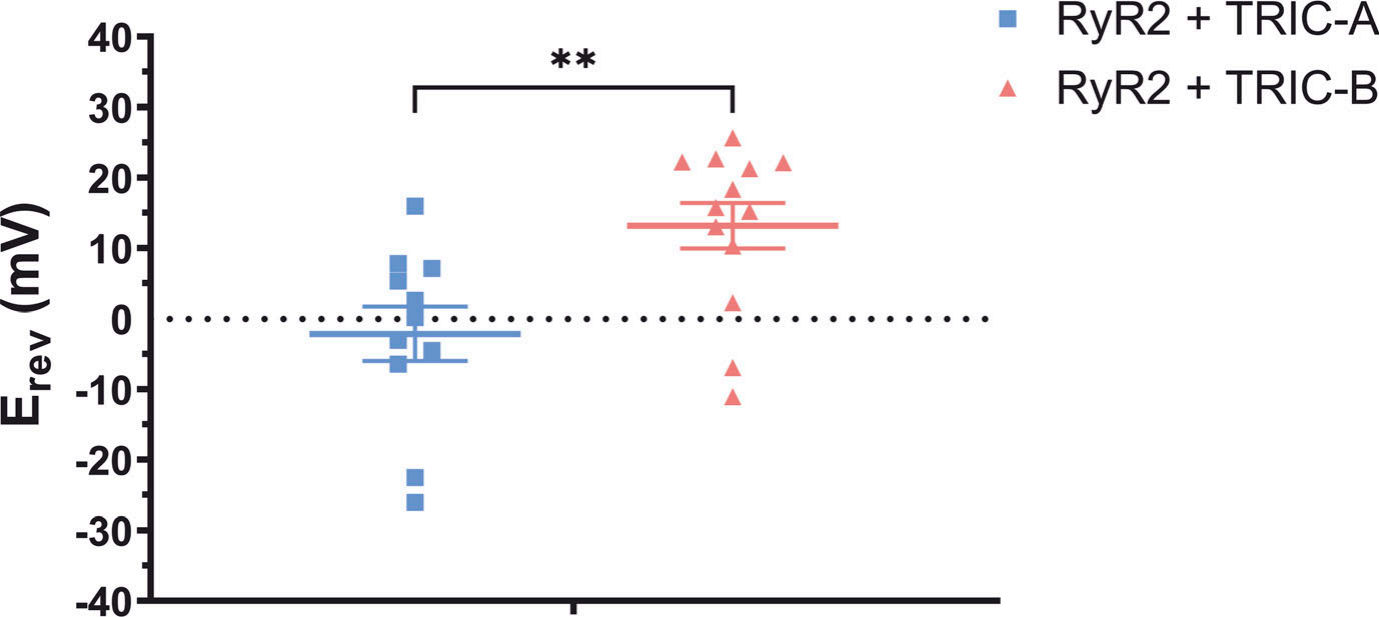
**Effect of coexpressing TRIC-A or TRIC-B with RyR2 on the reversal potential.** The mean E_rev_ values obtained after the application of a voltage ramp are compared for RyR2+TRIC-A (blue squares; *n* =11) and RyR2+TRIC-B (red triangles; *n* = 13) vesicle preparations. Ramp currents were recorded in triplicates for each bilayer experiment and the three E_rev_s measured were averaged and plotted as a single data point. An unpaired *t* test gives P = 0.0055, indicated by **. Symbols indicate values from individual experiments and bars indicate mean ± SEM.

Fig. 9 shows the E_rev_ values obtained from several experiments so that a mean value can be found for the RyR2+TRIC-A and RYR2+TRIC-B cells. It can be seen that there is a significant difference between the E_rev_ value for TRIC-A and that for TRIC-B. The E_rev_ values indicate that both TRIC-A and TRIC-B exhibit some permeability to K^+^ but that TRIC-B is more permeable to Cl^−^ than to K^+^ since the E_rev_ is closer to the E_rev_ for a channel ideally selective to Cl^−^ (+32 mV, the calculated E_rev_ for KCl in the 740 mM KCl cytosolic:210 mM KCl luminal solutions).

**Figure 10.**
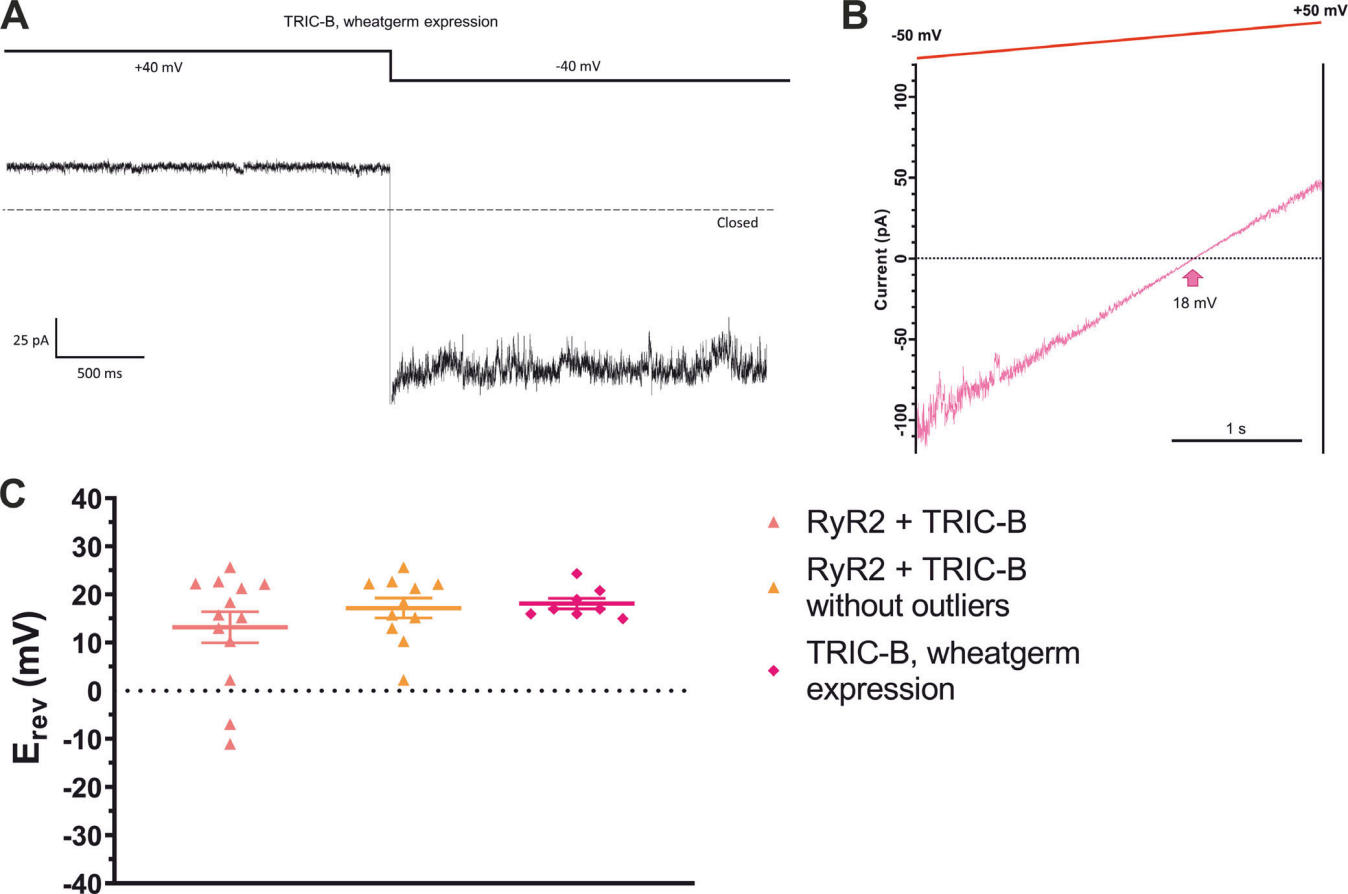
**Example of single-channel recordings of TRIC-B expressed in wheat germ. (A)** Liposomes containing TRIC-B expressed in the wheat germ system were fused into bilayers under KCl gradient conditions (740 mM KCl *cis*; 210 mM KCl *trans*), 2 µM free Ca^2+^. The recording illustrates the typical multiple-channel fluctuations observed in the KCl gradient and at holding potentials of +40 and −40 mV. The dashed line represents the zero current level. The large current observed at −40 mV indicates that TRIC-B is predominantly permeable to Cl^−^ ions. **(B)** Example of current fluctuations of TRIC-B from wheat germ during the application of a voltage ramp under gradient (pink trace) conditions. E_rev_ is indicated by the arrow. **(C)** The mean E_rev_ values for each bilayer are shown for RyR2+TRIC-B (red triangles; *n* = 13; data from Fig. 9), RyR2+TRIC-B without outliers (orange triangles; *n* = 11) and TRIC-B expressed in wheat germ (pink squares; *n* = 8). One-way ANOVA shows no significant difference with P value = 0.3678.

These results are consistent with ramp currents we obtained using the wheat germ expression system, as shown in Fig. 10. The important advantage this expression system conferred is that we were able to express and purify TRIC-B without the use of any detergents (Fig. S3). In Fig. 10 A, the large current at −40 mV indicates that TRIC-B is predominantly permeable to Cl^−^. Fig. 10 B shows a typical example of a ramp current with the E_rev_ indicated by the pink arrow, again showing that TRIC-B is more permeable to Cl^−^ than K^+^. Fig. 10 C shows the mean of individual E_rev_ values we measured using this method and compared with the E_rev_ measurements of TRIC-B obtained from expressing TRIC-B in HEK cells. This data confirms that TRIC-B exhibits a similar E_rev_ irrespective of the expression system used and that it is primarily permeable to Cl^−^.

Additionally, these results demonstrate that the TRIC-B channels from wheat germ where no RyR2 channels are present show highly similar permeability properties to TRIC-B expressed together with RyR2 in HEK cells. Therefore, these results suggest that the presence of RyR2 is not necessary for fully functional TRIC channels.

## Discussion

Our experiments provide novel evidence that TRIC-A and TRIC-B interact with RyR2 channels to alter RyR2 gating and also suggest that TRIC channels may be non-selective ion channels, permeable to both anions and cations.

Coexpression of TRIC-A or TRIC-B together with RyR2 in HEK293 cells does not affect the single-channel conductance of RyR2 when monitored after incorporation into bilayers but does affect channel gating suggesting that both TRIC-A and TRIC-B bind to RyR2. At −30 mV, when current is flowing in the luminal to cytosolic direction, the presence of TRIC-A significantly potentiates the ability of cytosolic Ca^2+^ to increase Po and this is particularly obvious at cytosolic [Ca^2+^] of 10 µM–1 mM. TRIC-B also exerts a potentiating effect although this is less marked. These results suggest a physical interaction between RyR2 and TRIC channels which was confirmed by our co-immunoprecipitation experiments (Fig. S1 B). Further examination of the effects on single-channel properties showed that the TRIC channels additionally affect the voltage dependence of RyR2 gating as steady-state RyR2 Po at +30 mV is lower than that at −30 mV at all cytosolic [Ca^2+^] if TRIC-A or TRIC-B is present, and inactivation of RyR2 by TRIC-B is more rapid than that of TRIC-A (Fig. 4 B). Voltage-dependent inactivation is a well-known property of RyR channels (Ma and Zhao, 1994; Percival et al., 1994; Sitsapesan et al., 1995; Laver and Lamb, 1998; Hill and Sitsapesan, 2002); however, it is not understood how it contributes to the physiological regulation of Ca^2+^ release during EC coupling. It has always been expected, since the Garcia and Miller report (Garcia and Miller, 1984), that there will be no large potential change across the SR during the Ca^2+^ release process because of the rapid flux of counter ions, and more recent experimentation in skeletal muscle has not contradicted this (Sanchez et al., 2018). The inactivation that we observe at +30 mV occurs as current is flowing in the cytosolic to luminal direction and so this could be a mechanism that prevents Ca^2+^ fluxing back into the SR via RyR2. Laver and Lamb (1998) suggest that RyR2 Po is the main driver of inactivation, but we would argue that certain ligands, for example 4,4′-diisothiocyanostilbene-2,2′-disulfonic acid (DIDS), can have an even greater effect (Hill and Sitsapesan, 2002). We also find that the voltage dependence of the native sheep cardiac RyR2 (Sitsapesan and Williams, 1994b) is not identical to that of the sheep-purified RyR2 (Hill and Sitsapesan, 2002). Thus, purification of RyR2 may alter the voltage dependence of Po, perhaps because critical binding proteins have been removed. It would not be unexpected, therefore, if voltage dependence of inactivation of recombinant RyR2 channels expressed in HEK cells differed from that of the native RyR2 derived from hearts if some proteins are missing in HEK cells. Much further work is required to understand the physiological relevance of this property of RyR channels. Taking all the TRIC-induced changes in RyR2 gating into consideration, overall, the presence of TRIC channels would be expected to enhance the opening of RyR2 at potentials when Ca^2+^ would flow from the SR lumen to the cytosol. Thus, the presence of either TRIC-A or TRIC-B might be expected to potentiate RyR2 activity and increase SR Ca^2+^ release during EC coupling, with the effect of TRIC-A being greater. Our results are thus in line with those of Zhou et al. (2020), where Ca^2+^ spark frequency was shown to be reduced in cardiomyocytes from *Tric-a* KO mice, and delayed onset of Ca^2+^ transients and time-to-peak amplitude of the transients were also observed.

If the single-channel properties of RyR2 can be regulated by the physical interaction with TRIC-A or -B, one might expect a reciprocal effect on the TRIC channels such that their correct functioning as ion channels depends on the presence of a Ca^2+^-release channel. Hence, the co-expressing of RyR2 with TRIC channels in HEK293 cells may provide a necessary stabilizing effect for TRIC channel function. Bilayer incorporation of membrane vesicles from HEK293 cells coexpressing TRIC-A or TRIC-B with RyR2 generally resulted in bilayers conducting more current than those where vesicles from HEK293 cells expressing only RyR2 were incorporated, as shown in the representative traces in Fig. 8. These currents were not due to RyR2 and were difficult to characterize. This is because the current amplitudes of the individual channel openings were small and difficult to accurately monitor because of the large noise of the bilayer system and because open and closing events were brief and hence not fully resolved. Recordings from previous reports of purified TRIC channel conductance and gating behavior also reveal these problems (Yazawa et al., 2007; Pitt et al., 2010; Su et al., 2017). Moreover, if TRIC channels gate into subconductance open states as these reports also suggest, then the reliable characterization of the gating and conductance properties of single TRIC channels becomes more problematic. Hence, it was our aim to incorporate multiple channels into bilayers to investigate if TRIC channels are selectively permeable to cations. Our findings that the E_rev_ of bilayers containing TRIC-A or TRIC-B was far from the E_rev_ expected for cation-selective channels (unless the gating of RyR2 channels dominated the total current flowing across the bilayer) suggests that the TRIC channels may be permeable to both K^+^ and Cl^−^. The results also suggest that TRIC-B channels may be more permeable to Cl^−^ than TRIC-A. These properties will still enable TRIC channels to be efficient pathways for counter-ion current across the SR. In fact, the ability to act as a pathway for both anions and cations would be expected to provide a more flexible counter-ion current that is suitable for a wider variety of cell types. In this regard, it is interesting that TRIC-B is present in many cell types (Yazawa et al., 2007), where perhaps the need for Cl^−^ flux is greater than that for K^+^ flux, whereas excitable cells tend to have high quantities of TRIC-A to support the larger SR Ca^2+^ release events that occur predominantly via RyR channels.

If both TRIC channels are permeable to Cl^−^, it suggests that neither TRIC-A nor TRIC-B is the SR K^+^ channel. For a long time, it has been suspected that the SR K^+^ channel could be one or both of the TRIC channels and this is because the irregular SR K^+^ channel subconductance state gating appeared similar to the current fluctuations caused by reconstituting purified TRIC channels into bilayers (Yazawa et al., 2007; Pitt et al., 2010; Su et al., 2017). When purified proteins are reconstituted into bilayers, there is always the possibility that the detergents or the purification procedures may alter protein function, thus giving rise to the multiple subconductance states. We did not, however, previously observe any opening events that were exactly the same conductance as the full openings of the SR K^+^ channel in 210 mM symmetrical KPIPES solutions (Pitt et al., 2010). The fully open-state single-channel conductance of the mouse skeletal SR K^+^ channel in these solutions is 208 ± 2 pS (*n* = 55; Eberhardt, 2018). In the current study, where we incorporated vesicles from HEK293 cells into bilayers without use of detergents or lengthy, harsh purification procedures, we still did not observe any channel events similar to the full opening events of the mouse SR K^+^ channel exemplified in Fig. 6. This data, coupled with the data suggesting that TRIC channels are non-selective ion channels, adds fuel to the idea that neither TRIC-A nor TRIC-B is the SR K^+^ channel. There is, however, another important consideration to be made where recombinant TRIC channels are prepared using artificial cDNA expression systems. X-ray crystals of TRIC channels demonstrate that various phospholipids are incorporated into the structure of the channels (Kasuya et al., 2016; Yang et al., 2016; Ou et al., 2017; Su et al., 2017; Wang et al., 2019). The position of the lipids varies with species and TRIC type but they are often embedded near or in the pore region and thus would be expected to affect single-channel conductance and ionic selectivity (Kasuya et al., 2016; Yang et al., 2016; Ou et al., 2017; Su et al., 2017; Wang et al., 2019). Not only must these lipids be available in sufficient quantities in the artificial expression system, but there may be cell-specific enzymes that are required for the building of the trimeric TRIC channels with their particular phospholipids in-built into the final structure. It is impossible to recreate, for example, the exact biochemical environment of striated muscle in an HEK293 cell, and so we must be cautious in trying to match TRIC channels to a particular ionic current observed in striated muscle SR membranes.

### Conclusions

In summary, our results indicate that TRIC-A and TRIC-B may exert complementary roles in directly regulating RyR2 gating during EC coupling. The presence of TRIC-A or TRIC-B alters RyR2 gating suggesting that both channels bind to RyR2. TRIC-A, however, exerts a greater stimulatory role than TRIC-B. Our results also highlight the need for further detailed biophysical examination of the gating and conductance properties of purified TRIC channels and the need for identification and characterization of all the Cl^−^ channels in muscle SR. If TRIC channels are indeed permeable to both K^+^ and Cl^−^ as our results suggest, this property will enable both TRIC-A and TRIC-B to provide counter-ion currents of K^+^ and Cl^−^ to balance Ca^2+^ movements across the SR, with TRIC-A providing a greater proportion of K^+^ current than TRIC-B. We hope that our study will direct further effort into the difficult task of characterizing the biophysical properties of TRIC channels in more detail and the mechanisms by which they affect RyR2 gating.

## Supplementary Material

Table S1 shows simple effects for the Po of type of HEK293 cell under different cytosolic [Ca^2+^] at a holding potential of −30 mV.

Table S2 shows simple effects for the Po of cytosolic [Ca^2+^] in different types of HEK293 cells at a holding potential of −30 mV.

Table S3 shows simple effects for the Po of type of HEK293 cell under different cytosolic [Ca^2+^] at a holding potential of +30 mV.

Table S4 shows simple effect of the Po of cytosolic [Ca^2+^] in different type of HEK293 cells at a holding potential of +30 mV.

SourceData FS1 is the source file for Fig. S1.
