# Supplementary material for: The biophysical properties of TRIC-A and TRIC-B and their interactions with RyR2
Source: J Gen Physiol. 2023 Sep 26;155(11):e202113070. doi: 10.1085/jgp.202113070 (PMC10522464; doi:10.1085/jgp.202113070)
Supplement: Table S1 — shows simple effects for the Po of type of HEK293 cell under different cytosolic [Ca2+] at a holding potential of −30 mV. [file JGP_202113070_TableS1.docx]

**Table S1. Simple effects for the Po of “type of HEK293 cell” under different cytosolic [Ca^2+^] at a holding potential of -30 mV**

| Po of RyR2 at -30 mV | Condition | Multiple comparisons test | Adjusted *p*-value (Sidak) |
| --- | --- | --- | --- |
|  | Ca^2+^ 2 μM | RyR2 +TRIC-A vs RyR2 only | 0.874 |
|  |  | RyR2 +TRIC-A vs. RyR2 +TRIC-B | 0.960 |
|  |  | RyR2 only vs. RyR2 +TRIC-B | 0.987 |
|  | Ca^2+^ 10 μM | RyR2 +TRIC-A vs. RyR2 only | **0.026** |
|  |  | RyR2 +TRIC-A vs. RyR2 +TRIC-B | **0.000155** |
|  |  | RyR2 only vs. RyR2 +TRIC-B | 0.460 |
|  | Ca^2+^ 100 μM | RyR2 +TRIC-A vs. RyR2 only | **0.0000000297** |
|  |  | RyR2 +TRIC-A vs. RyR2 +TRIC-B | **0.000557** |
|  |  | RyR2 only vs. RyR2 +TRIC-B | **0.00532** |
|  | Ca^2+^ 1 mM | RyR2 +TRIC-A vs. RyR2 only | **0.00291** |
|  |  | RyR2 +TRIC-A vs. RyR2 +TRIC-B | 0.059 |
|  |  | RyR2 only vs. RyR2 +TRIC-B | 0.348 |
|  | Ca^2+^ 2 mM | RyR2 +TRIC-A vs. RyR2 only | **0.00251** |
|  |  | RyR2 +TRIC-A vs. RyR2 +TRIC-B | **0.00624** |
|  |  | RyR2 only vs. RyR2 +TRIC-B | 0.842 |
