# Supplementary material for: The biophysical properties of TRIC-A and TRIC-B and their interactions with RyR2
Source: J Gen Physiol. 2023 Sep 26;155(11):e202113070. doi: 10.1085/jgp.202113070 (PMC10522464; doi:10.1085/jgp.202113070)
Supplement: Table S2 — shows simple effects for the Po of cytosolic [Ca2+] in different types of HEK293 cells at a holding potential of −30 mV. [file JGP_202113070_TableS2.docx]

**Table S2. Simple effects for the Po of “cytosolic [Ca^2+^]” in different types of HEK293 cells at a holding potential of -30 mV**

| Po of RyR2 at -30 mV | Condition | Multiple comparisons test | Adjusted *p*-value (Sidak) |
| --- | --- | --- | --- |
|  | RyR2 only | Ca^2+^ 2 μM vs. Ca^2+^ 10 μM | 0.109 |
|  |  | Ca^2+^ 2 μM vs. Ca^2+^ 100 μM | 0.996 |
|  |  | Ca^2+^ 2 μM vs. Ca^2+^ 1 mM | 1.000 |
|  |  | Ca^2+^ 2 μM vs. Ca^2+^ 2 mM | 0.992 |
|  |  | Ca^2+^ 10 μM vs. Ca^2+^ 100 μM | 0.300 |
|  |  | Ca^2+^ 10 μM vs. Ca^2+^ 1 mM | **0.008** |
|  |  | Ca^2+^ 10 μM vs. Ca^2+^ 2 mM | **0.012** |
|  |  | Ca^2+^ 100 μM vs. Ca^2+^ 1 mM | 0.966 |
|  |  | Ca^2+^ 100 μM vs. Ca^2+^ 2 mM | 0.538 |
|  |  | Ca^2+^ 1 mM vs. Ca^2+^ 2 mM | 1.000 |
|  | RyR2 +TRIC-A | Ca^2+^ 2 μM vs. Ca^2+^ 10 μM | **0.0000613** |
|  |  | Ca^2+^ 2 μM vs. Ca^2+^ 100 μM | **0.0000000000416** |
|  |  | Ca^2+^ 2 μM vs. Ca^2+^ 1 mM | 0.068 |
|  |  | Ca^2+^ 2 μM vs. Ca^2+^ 2 mM | **0.030** |
|  |  | Ca^2+^ 10 μM vs. Ca^2+^ 100 μM | **0.00907** |
|  |  | Ca^2+^ 10 μM vs. Ca^2+^ 1 mM | 0.357 |
|  |  | Ca^2+^ 10 μM vs. Ca^2+^ 2 mM | 0.897 |
|  |  | Ca^2+^ 100 μM vs. Ca^2+^ 1 mM | **0.000376** |
|  |  | Ca^2+^ 100 μM vs. Ca^2+^ 2 mM | **0.00162** |
|  |  | Ca^2+^ 1 mM vs. Ca^2+^ 2 mM | 1.000 |
|  | RyR2 +TRIC-B | Ca^2+^ 2 μM vs. Ca^2+^ 10 μM | 0.941 |
|  |  | Ca^2+^ 2 μM vs. Ca^2+^ 100 μM | **0.0000103** |
|  |  | Ca^2+^ 2 μM vs. Ca^2+^ 1 mM | 0.948 |
|  |  | Ca^2+^ 2 μM vs. Ca^2+^ 2 mM | 1.000 |
|  |  | Ca^2+^ 10 μM vs. Ca^2+^ 100 μM | **0.0000600** |
|  |  | Ca^2+^ 10 μM vs. Ca^2+^ 1 mM | 1.000 |
|  |  | Ca^2+^ 10 μM vs. Ca^2+^ 2 mM | 0.704 |
|  |  | Ca^2+^ 100 μM vs. Ca^2+^ 1 mM | **0.002** |
|  |  | Ca^2+^ 100 μM vs. Ca^2+^ 2 mM | **0.0000000159** |
|  |  | Ca^2+^ 1 mM vs. Ca^2+^ 2 mM | 0.780 |
