# Supplementary material for: The biophysical properties of TRIC-A and TRIC-B and their interactions with RyR2
Source: J Gen Physiol. 2023 Sep 26;155(11):e202113070. doi: 10.1085/jgp.202113070 (PMC10522464; doi:10.1085/jgp.202113070)
Supplement: Table S3 — shows simple effects for the Po of type of HEK293 cell under different cytosolic [Ca2+] at a holding potential of +30 mV. [file JGP_202113070_TableS3.docx]

**Table S3. Simple effects for the Po of “type of HEK293 cell” under different cytosolic [Ca^2+^] at a holding potential of +30 mV**

| Po of RyR2 at +30 mV | Condition | Multiple comparisons test | Adjusted *p*-value (Sidak) |
| --- | --- | --- | --- |
|  | Ca^2+^ 2 μM | RyR2 only vs. RyR + TRIC-A | 1.000 |
|  |  | RyR2 only vs. RyR2 + TRIC-B | 0.999 |
|  |  | RyR + TRIC-A vs. RyR2 + TRIC-B | 0.999 |
|  | Ca^2+^ 10 μM | RyR2 only vs. RyR + TRIC-A | **0.00295** |
|  |  | RyR2 only vs. RyR2 + TRIC-B | **0.000311** |
|  |  | RyR + TRIC-A vs. RyR2 + TRIC-B | 0.941 |
|  | Ca^2+^ 100 μM | RyR2 only vs. RyR + TRIC-A | 0.301 |
|  |  | RyR2 only vs. RyR2 + TRIC-B | **0.048** |
|  |  | RyR + TRIC-A vs. RyR2 + TRIC-B | 0.904 |
|  | Ca^2+^ 1 mM | RyR2 only vs. RyR + TRIC-A | 0.956 |
|  |  | RyR2 only vs. RyR2 + TRIC-B | 0.956 |
|  |  | RyR + TRIC-A vs. RyR2 + TRIC-B | 1.000 |
|  | Ca^2+^ 2 mM | RyR2 only vs. RyR + TRIC-A | 0.947 |
|  |  | RyR2 only vs. RyR2 + TRIC-B | 0.939 |
|  |  | RyR + TRIC-A vs. RyR2 + TRIC-B | 1.000 |
