# Supplementary material for: The biophysical properties of TRIC-A and TRIC-B and their interactions with RyR2
Source: J Gen Physiol. 2023 Sep 26;155(11):e202113070. doi: 10.1085/jgp.202113070 (PMC10522464; doi:10.1085/jgp.202113070)
Supplement: Table S4 — shows simple effect of the Po of cytosolic [Ca2+] in different type of HEK293 cells at a holding potential of +30 mV. [file JGP_202113070_TableS4.docx]

**Table S4. Simple effect of the Po of“cytosolic [Ca^2+^]” in different type of HEK293 cells at a holding potential of +30 mV**

| Po of RyR2 at +30 mV | Condition | Tukey's multiple comparisons test | Adjusted *p*-value (Sidak) |
| --- | --- | --- | --- |
|  | RyR2 only | Ca^2+^ 2 μM vs. Ca^2+^ 10 μM | **0.000103** |
|  |  | Ca^2+^ 2 μM vs. Ca^2+^ 100 μM | 0.459 |
|  |  | Ca^2+^ 2 μM vs. Ca^2+^ 1 mM | 1.000 |
|  |  | Ca^2+^ 2 μM vs. Ca^2+^ 2 mM | 1.000 |
|  |  | Ca^2+^ 10 μM vs. Ca^2+^ 100 μM | **0.0383** |
|  |  | Ca^2+^ 10 μM vs. Ca^2+^ 1 mM | **0.00000551** |
|  |  | Ca^2+^ 10 μM vs. Ca^2+^ 2 mM | **0.000103** |
|  |  | Ca^2+^ 100 μM vs. Ca^2+^ 1 mM | 0.236 |
|  |  | Ca^2+^ 100 μM vs. Ca^2+^ 2 mM | 0.088 |
|  |  | Ca^2+^ 1 mM vs. Ca^2+^ 2 mM | 1.000 |
|  | RyR + TRIC-A | Ca^2+^ 2 μM vs. Ca^2+^ 10 μM | 1.000 |
|  |  | Ca^2+^ 2 μM vs. Ca^2+^ 100 μM | 1.000 |
|  |  | Ca^2+^ 2 μM vs. Ca^2+^ 1 mM | 0.998 |
|  |  | Ca^2+^ 2 μM vs. Ca^2+^ 2 mM | 0.981 |
|  |  | Ca^2+^ 10 μM vs. Ca^2+^ 100 μM | 1.000 |
|  |  | Ca^2+^ 10 μM vs. Ca^2+^ 1 mM | 0.974 |
|  |  | Ca^2+^ 10 μM vs. Ca^2+^ 2 mM | 0.991 |
|  |  | Ca^2+^ 100 μM vs. Ca^2+^ 1 mM | 0.999 |
|  |  | Ca^2+^ 100 μM vs. Ca^2+^ 2 mM | 0.993 |
|  |  | Ca^2+^ 1 mM vs. Ca^2+^ 2 mM | 0.993 |
|  | RyR2 +TRIC-B | Ca^2+^ 2 μM vs. Ca^2+^ 10 μM | 1.000 |
|  |  | Ca^2+^ 2 μM vs. Ca^2+^ 100 μM | 0.999 |
|  |  | Ca^2+^ 2 μM vs. Ca^2+^ 1 mM | 0.999 |
|  |  | Ca^2+^ 2 μM vs. Ca^2+^ 2 mM | 0.985 |
|  |  | Ca^2+^ 10 μM vs. Ca^2+^ 100 μM | 1.000 |
|  |  | Ca^2+^ 10 μM vs. Ca^2+^ 1 mM | 1.000 |
|  |  | Ca^2+^ 10 μM vs. Ca^2+^ 2 mM | 1.000 |
|  |  | Ca^2+^ 100 μM vs. Ca^2+^ 1 mM | 1.000 |
|  |  | Ca^2+^ 100 μM vs. Ca^2+^ 2 mM | 1.000 |
|  |  | Ca^2+^ 1 mM vs. Ca^2+^ 2 mM | 1.000 |
