# Supplementary material for: The biophysical properties of TRIC-A and TRIC-B and their interactions with RyR2
Source: J Gen Physiol. 2023 Sep 26;155(11):e202113070. doi: 10.1085/jgp.202113070 (PMC10522464; doi:10.1085/jgp.202113070)
Supplement: SourceData FS1 — is the source file for Fig. S1. [file JGP_202113070_SourceDataFS1.pdf]

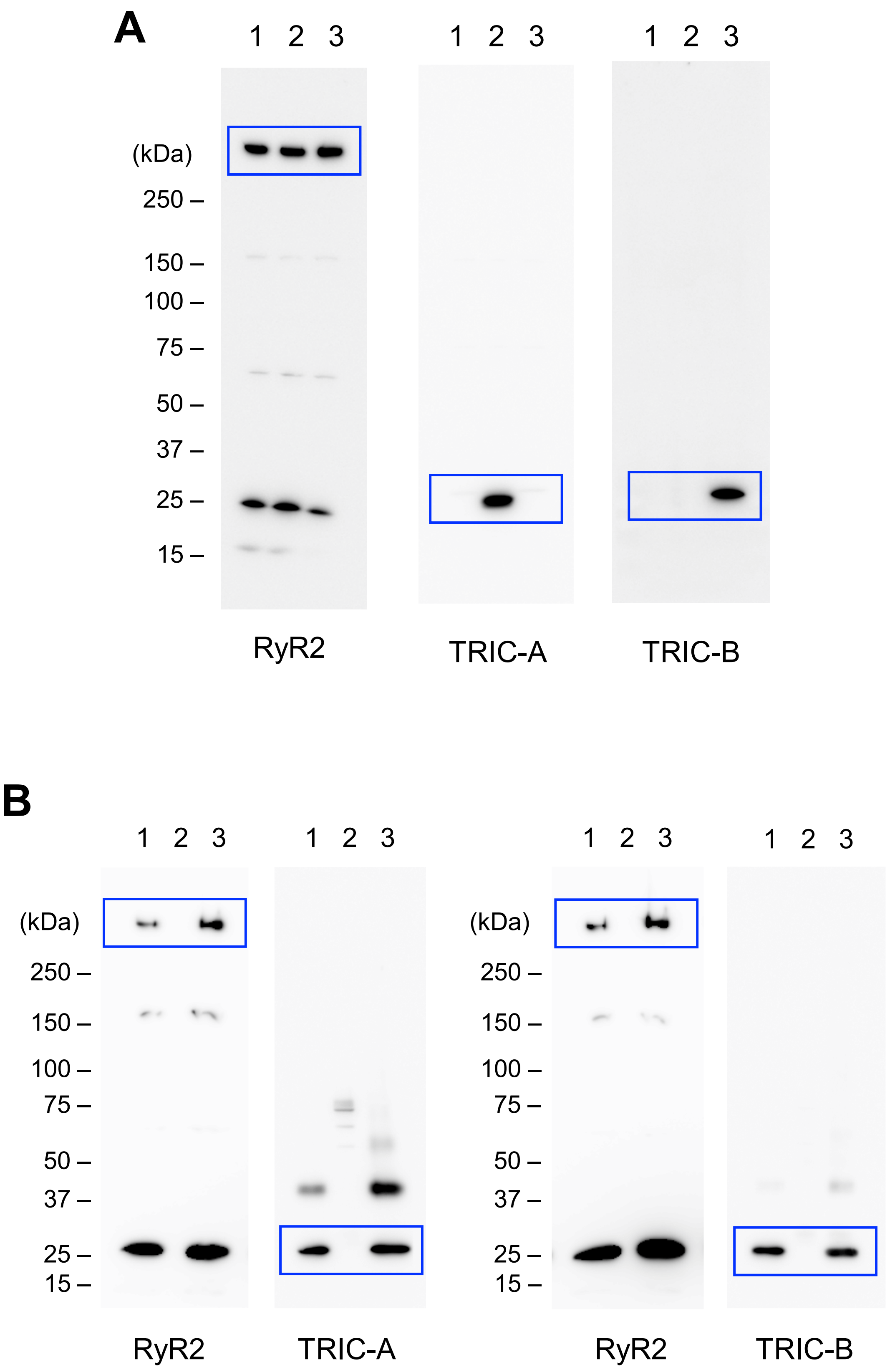

SourceDataFS1. (A) Western blots of the microsomal membrane vesicles isolated from RyR2-expressing HEK293 cells that were either uninfected (lane 1), infected with TRIC-A baculovirus (BV) (lane 2) or TRIC-B BV (lane 3). **(B)** Western blots of lysate (lane 1), the immunoprecipitated product by control IgG (lane 2) and the immunoprecipitated product by anti-SBP antibody (lane 3).
